# CRISPR-Cas9-based precise engineering of SlHyPRP1 protein towards multi-stress tolerance in tomato

**DOI:** 10.3389/fpls.2023.1186932

**Published:** 2023-05-15

**Authors:** Mil Thi Tran, Geon Hui Son, Young Jong Song, Ngan Thi Nguyen, Seonyeong Park, Thanh Vu Thach, Jihae Kim, Yeon Woo Sung, Swati Das, Dibyajyoti Pramanik, Jinsu Lee, Ki-Ho Son, Sang Hee Kim, Tien Van Vu, Jae-Yean Kim

**Affiliations:** ^1^ Division of Applied Life Science (BK21 Four Program), Plant Molecular Biology and Biotechnology Research Center, Gyeongsang National University, Jinju, Republic of Korea; ^2^ Crop Science and Rural Development Division, College of Agriculture, Bac Lieu University, Bac Lieu, Vietnam; ^3^ Division of Horticultural Science, Gyeongsang National University, Jinju, Republic of Korea; ^4^ Division of Life Science, Gyeongsang National University, Jinju, Republic of Korea; ^5^ National Key Laboratory for Plant Cell Biotechnology, Agricultural Genetics Institute, Hanoi, Vietnam; ^6^ Nulla Bio R&D Center, Nulla Bio Inc., Jinju, Republic of Korea

**Keywords:** CRISPR-Cas9, HyPRP1, abiotic stress, biotic stress, heat stress tolerance, multi-stress tolerance

## Abstract

Recently, CRISPR-Cas9-based genome editing has been widely used for plant breeding. In our previous report, a tomato gene encoding hybrid proline-rich protein 1 (HyPRP1), a negative regulator of salt stress responses, has been edited using a CRISPR-Cas9 multiplexing approach that resulted in precise eliminations of its functional domains, proline-rich domain (PRD) and eight cysteine-motif (8CM). We subsequently demonstrated that eliminating the PRD domain of HyPRP1 in tomatoes conferred the highest level of salinity tolerance. In this study, we characterized the edited lines under several abiotic and biotic stresses to examine the possibility of multiple stress tolerance. Our data reveal that the 8CM removal variants of HK and the KO alleles of both HK and 15T01 cultivars exhibited moderate heat stress tolerance. Similarly, plants carrying either the domains of the PRD removal variant (PR1v1) or 8CM removal variants (PR2v2 and PR2v3) showed better germination under osmosis stress (up to 200 mM mannitol) compared to the WT control. Moreover, the PR1v1 line continuously grew after 5 days of water cutoff. When the edited lines were challenged with pathogenic bacteria of *Pseudomonas syringae* pv. *tomato* (*Pto*) DC3000, the growth of the bacterium was significantly reduced by 2.0- to 2.5-fold compared to that in WT plants. However, the edited alleles enhanced susceptibility against *Fusarium oxysporum* f. sp. *lycopersici*, which causes fusarium wilt. CRISPR-Cas9-based precise domain editing of the *SlHyPRP1* gene generated multi-stress-tolerant alleles that could be used as genetic materials for tomato breeding.

## Introduction

Genome editing (GE) technology using CRISPR-Cas nucleases has recently emerged as a revolutionary plant breeding technology and shows promise to the agriculture industry (Belhaj et al., 2013; Chen et al., 2019; Vu et al., 2022). CRISPR-Cas-based GE has been effectively applied for plant breeding at high precision levels (Chen et al., 2019). It has been successful in modifying tomato genomes and precision breeding to create new alleles, not including linkage drag (Zsögön et al., 2017; Li et al., 2018b; Zsögön et al., 2018; Vu et al., 2020a).

Recently, unforeseen climate changes have caused many biotic and abiotic stresses that negatively affect multiple aspects of tomato production, such as yield and quality, thereby threatening agriculture sustainability. Therefore, new tomato cultivars capable of biotic and abiotic stress tolerance are important subjects of breeding using GE technology. The application of CRISPR-Cas9 in tomato genome engineering obtained targeted mutations at four different loci. Subsequently, stable inheritance of the edited alleles in the next generations was observed through genotyping and phenotyping (Brooks et al., 2014). CRISPR-Cas-based targeted mutants of CLAVATA/WUS showed different ranges in tomato shoot meristem size (Fletcher, 2018). Moreover, CRISPR-Cas9 or/and CRISPR-Cas12a were employed to effectively edit genes for obtaining enhanced abiotic stress tolerance in various plant species such as rice and wheat (Shan et al., 2013; Shan et al., 2014; Endo et al., 2016; Osakabe et al., 2018), tomato (Brooks et al., 2014; Tran et al., 2020; Vu et al., 2020b), and apple (Malnoy et al., 2016; Nishitani et al., 2016; Osakabe et al., 2018; Charrier et al., 2019). In particular, they have been successfully applied to tomatoes for drought tolerance (Wang et al., 2017a; Li et al., 2019), salt tolerance (Tran et al., 2020; Vu et al., 2020b; Wang et al., 2021), and chilling and heat tolerance (Li et al., 2018a; Yin et al., 2018).

Proline-rich proteins (PRPs) are involved in cell-wall signaling, plant development, and stress responses (Kavi Kishor et al., 2015). Hybrid proline-rich proteins (HyPRPs) comprise an N-terminal hydrophobic signal peptide and two different domains: a repetitive proline-rich domain (PRD) in the N-terminus and a C-terminal 8-cysteine motif (8CM) domain. The conserved amino acid sequences from tomato HyPRP1 retrieval from an alignment with its homologs in various plant species (Yeom et al., 2012) are cell-wall glycoproteins enriched with proline and are also known to function as cell-wall structural proteins. In addition, *HyPRP1* is a crucial gene of PRP and 8CM protein families that might play a critical role in multiple stress responses in tomatoes (Li et al., 2016). HyPRP1 was shown to have diverse functions for stress responses of different plant species. HyPRP1 functioned as a cell death positive regulator and a negative regulator of biotic stress defense in pepper (*Capsicum annuum*) and tobacco (*Nicotiana benthamiana*) (Yeom et al., 2012). In contrast, a report on cotton indicated that GbHyPRP1 plays a negative role in controlling cotton resistance to the vascular wilt disease caused by *V. dahlia.* The RNAi-mediated *GbHyPRP1* silencing lines showed thicker and composition-altered cell walls and enhanced reactive oxygen species (ROS) accumulation, possibly resulting in enhanced cotton resistance to *V. dahlia* (Yang et al., 2018). Moreover, overexpression of a pigeon pea HyPRP (CcHyPRP) gene induced abiotic stress tolerance in yeast, *Arabidopsis* (Priyanka et al., 2010), and rice (Mellacheruvu et al., 2016). In tomatoes, SlHyPRP1 was shown to be negatively involved in multi-stress responses. RNAi-mediated *SlHyPRP1* silencing resulted in enhanced stress (oxidative stress, dehydration, and salinity) tolerance without phenotypic alteration (Li et al., 2016).

In a previous report, we generated precisely edited alleles that included eliminating either the PRD or 8CM domains of the tomato HyPRP1 protein and completely deleting the domains (Tran et al., 2020). We showed that several alleles carrying PRD or 8CM or without PRD-8CM domains generated by multiplexed CRISPR-Cas9-based GE tools in two local tomato varieties conferred salinity tolerance. In this study, we report data showing multiple stress tolerance enabled by the *SlHyPRP1*-edited lines in extensive tests using biotic and abiotic stresses. Our data reveal the importance of *HyPRP1* editing in breeding multiple stress-tolerant crops.

## Results

### Preparation of homozygous seeds carrying the *SlHyPRP1*-edited alleles

In our previous work (Tran et al., 2020), we used the multiplexed CRISPR-Cas9 approach to generate different truncated versions of SlHyPRP1 without either the domain or both (**Figures S1**, **S2**). Our data revealed the critical role of PRD as a negative regulator in salinity response. This study was designed to further characterize the precisely edited alleles (**Supplementary Table S1**) in response to other biotic and abiotic stresses for examining alleles that can confer multiple-stress tolerance. To do that, we harvested seeds of the lines that show homozygosity of the edited alleles. The inheritance of these edited alleles of HK and 15T01 cultivars in the next generation as homozygous genotypes was first validated (**Supplementary Figures S3**, **S4**), and the seeds of several plants among them were subsequently subjected to stress tolerance assessment.

### *SlHyPRP1*-edited lines exhibited heat stress tolerance

#### PRD and complete KO lines exhibit heat stress tolerance in the HK variety

**Figure 1:**
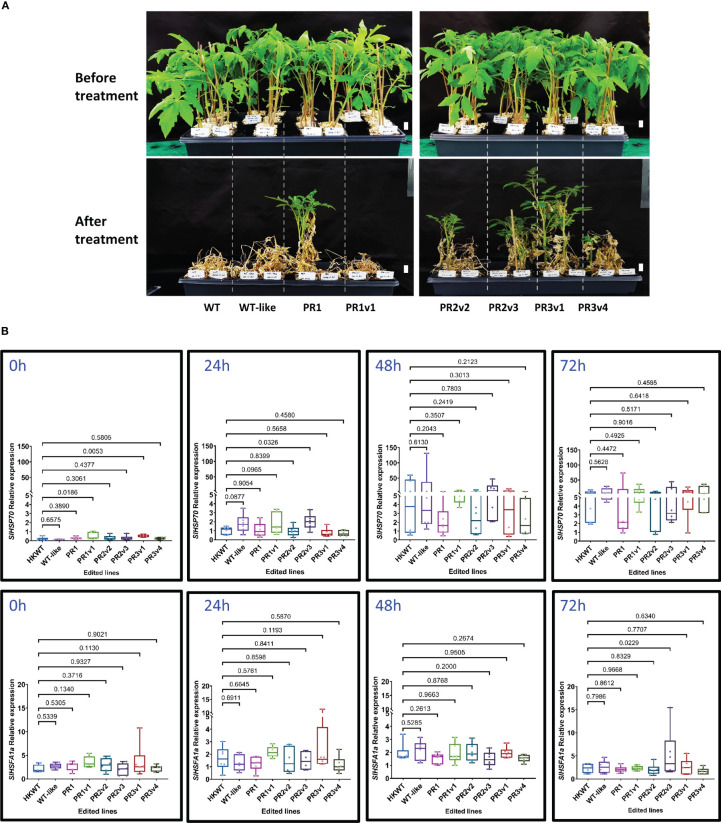
Heat stress tolerance exhibited by the SlHyPRP1 precisely edited HK lines. **(A)** Plants before treatment and at 30 days post-recovery. Six-week-old seedlings were subjected to high-temperature conditions for 6 days (3 days at 42°C and then 3 days at 45°C) under 16 h light/8 h dark in the growth chamber. They were then moved to 25°C (16 h light/8 h dark) conditions for recovery. Photographs were taken at 1-month post-recovery. The names of plants/lines are denoted at the bottom of each panel. Scale bars: 2 cm. **(B)** Box plots showing relative transcript levels of *SlHSP70* and *SlHSFA1a* of the heat-treated plants quantified by qRT-PCRs. Leaf tissues of the plants were collected at 0, 24, 48, and 72 h of heat treatment. Data were collected from six biological replicates. The multiple comparisons and illustrations of the relative expression levels were described in the *Materials and methods* section. The *p*-value is shown on the top of the compared boxes. The error bars indicate ± SEM. WT: wild-type HK cultivar; WT-like: segregated WT siblings of the edited plants; PR1: precise edited event PR1; PR1v1: PR1 variant; PR2v2: variant 2 of precise edited event PR2; PR2v3: variant 3 of precise edited event PR2; PR3v1: variant 1 of precise edited event PR3; PR3v4: variant 4 of precise edited event PR3.

Heat stress causes the suppression of photosynthesis, leading to growth defects and reduced productivity (Fahad et al., 2017). Thus, the heat-tolerant trait is in great demand in crop plants. We initially treated the HyPRP-allele-carrying plants with high-temperature conditions as described by Yu et al. (2019) with minor modifications. We found that the lines carrying alleles missing either the 8CM (PR1) or PRD (PR2v2) domain exhibited moderate heat tolerance, while the KO (PR3v1 and PR3v4) lines exhibited strong heat tolerance (**Figure 1A**, **Supplementary Figure S5**). The PR1 line appeared tolerant to the treatment. However, the other treatments could not repeat the responses (**Supplementary Figure S5**), indicating that its associated allele could not be strong enough to provide a durable heat tolerance.

Several genes (*HSP70* and *HSFA1a*) commonly known to be involved in heat responses were analyzed at the transcriptional level from the heat-treated plants. HSP70 was shown to be one of the critical molecular chaperones in plants that help to maintain and restore protein homeostasis, which is necessary for plant survival under heat stress (Hahn et al., 2011), and HSFA1a is a marker for thermotolerance (Liu et al., 2013). Although the *SlHSP70* relative expression level of precise PRD removal variant (PR1) and knockout (PR3v1) lines are significantly higher than that in WT before treatment, they are not very different during heat treatment (**Figure 1B**). Post-heat treatment, the *SlHSP70* and *SlHSFA1a* relative expression levels are also not significantly different among the edited lines and WT, except the PR2v3 line that showed higher levels of *SlHSP70* mRNAs at 24 h (*p*-value = 0.0326) and 72 h (*p* = 0.0229), respectively (**Figure 1B**). These data indicate that most of the edited alleles do not directly affect the transcription of the *SlHSP70* and *SlHSFA1a* genes. In the case of the line PR2v3, enhancing *SlHSFA1a* mRNA levels at 24 h and 72 h might not be sufficient to elaborate its heat tolerance.

In addition, we also evaluate transcript levels of a ROS-scavenging enzyme (SlZn/CuSOD) during the heat treatments. Unexpectedly, there is no significant difference in the transcription of *SlZn/CuSOD* between all edited lines and the WT and WT-like controls before and after 72 h heat treatment (**Supplementary Figure S6**). Furthermore, ROS accumulation, including singlet oxygen ( ${\text{O}}_{2}^{-}$ ) and hydrogen peroxide (H_2_O_2_), detected using NBT and DAB staining in tomato leaves, respectively, was moderately reduced in most edited lines after heat treatment. In contrast, the ROS levels were enhanced in the WT and WT-like controls (**Supplementary Figure S7**). These data indicate that heat tolerance was partially achieved by the enhanced expression of *SlHSP70* before heat treatment and efficient ROS scavenging in the edited plants during heat treatment (**Supplementary Figure S7**).

#### Complete KO lines of the 15T01 variety tolerated heat stress

**Figure 2:**
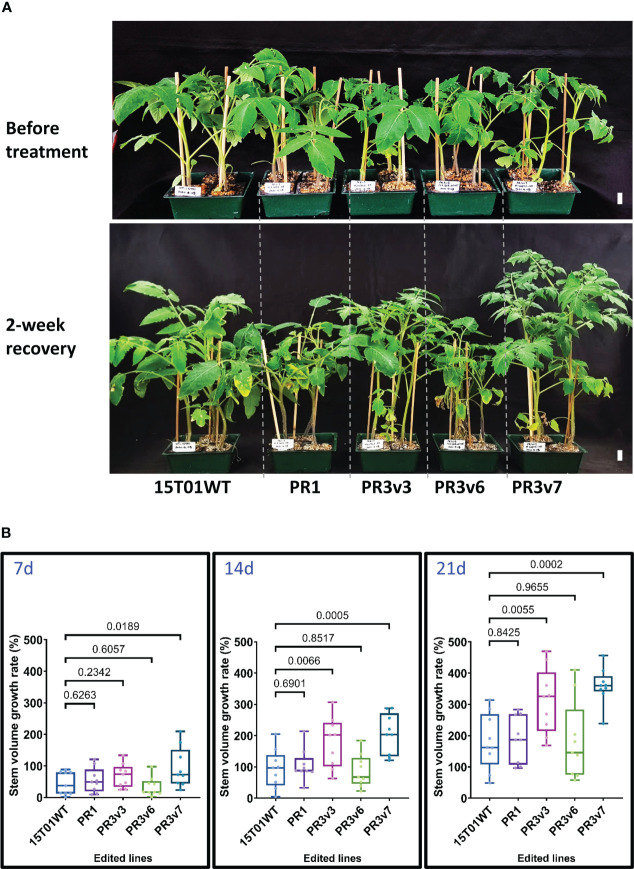
Heat stress tolerance exhibited by the edited 15T01 lines. **(A)** Plants before treatment and at 30 days post-recovery. Five-week-old seedlings were subjected to a 42°C (16 h light/8 h dark) chamber for 16 h and then moved to 25°C (16 h light/8 h dark) in the culture room. Photographs were taken before treatment and at 2 weeks post-recovery. The name of plants/lines are denoted at the bottom of each panel. The horizontal lines indicate plants from the same, edited lines. Scale bars: 2 cm. **(B)** The growth rate represented by the stem volume of the edited lines was recorded at 7, 14, and 21 days post-recovery compared to the parental plants under heat stress. Data were collected in nine biological replicates. Statistical analysis is detailed in the method. The *p*-value is shown on the top of the compared boxes. The error bars are ± SEM. 15T01WT: wild-type 15T01 cultivar, PR1: precise edited event PR1, PR3v3: variant 3 of precise edited event PR3, PR3v6: variant 6 of precise edited event PR3, PR3v7: variant 6 of precise edited event PR3.

To further determine whether the edited *SlHyPRP1* lines obtained with the 15T01 variety also enhanced heat tolerance, 5-week-old plants (15T01 cultivar) of edited lines consisting of one precise PRD removal (PR1) and three knockout (PR3v3, PR3v6, and PR3v7) lines (**Supplementary Table S1**, **Figure S4**) were subjected to a shorter period of 42°C incubation for only 16 h. Accordingly, sampling for qRT-PCRs was done at 0, 1, 8, and 16 h. After 2-week recovery, lines (PR3v3 and PR3v7) carrying KO alleles exhibited significantly higher recovery rates than the WT control, indicating that they were heat-tolerant. In contrast, PR1 and PR3v6 lines are more heat-sensitive (**Figure 2**, **Supplementary Figure S8**). Again, in the 15T01 genetic background, the plants missing the PRD domain (PR1) showed similar heat responses to that of the HK. Unfortunately, we could not obtain the 8CM removal allele with the 15T01 variety and, hence, cannot conclude the role of that allele in the 15T01 background. These data also suggest that the milder heat treatment may be better for assessing the heat tolerance levels of the edited lines.

**Figure 3:**
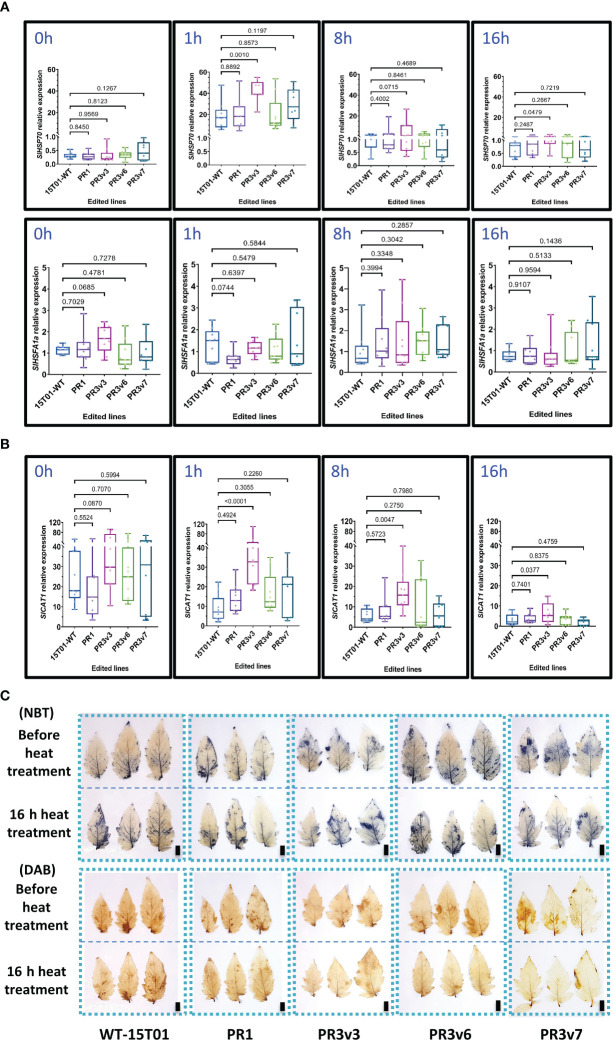
Relative expression of genes related to heat stress responses. **(A)** Relative transcript levels of *SlHSP70* and *SlHSFA1a* of the heat-treated 15T01 lines quantified by qRT-PCRs. **(B)** The relative transcript levels of *SlCAT1* of the heat-treated 15T01 lines were quantified by qRT-PCRs. **(C)** Effects of the 15T01-based *SlHyPRP1* edited lines on ROS production (NBT and DAB staining) under heat stress. Leaves of the heat-treated plants were collected before and after 16 h of the heat treatment and stained by the ROS-specific dyes. The top panel of each staining type represents leaves grown at 25°C before heat treatment, and the bottom panel represents leaves of the same plants treated at 42°C for 16 h. In **(A, B)**, leaf tissues of the plants were collected at 0, 1, 8, and 16 h of heat treatment. Data were collected from nine biological replicates. Statistical analysis of the data and plotting follows the method description. The *p*-value is shown on the top of the compared boxes. 15T01WT: wild-type 15T01 cultivar, PR1: precise edited event PR1, PR3v3: variant 3 of precise edited event PR3, PR3v6: variant 6 of precise edited event PR3, PR3v7: variant 6 of precise edited event PR3. Scale bars: 1 cm.

Similarly, *SlHSP70* and *SlHSFA1a* transcript levels were also analyzed in these experiments. Before treatment, both *SlHSP70* and *SlHSFA1a* relative expression levels of all edited lines and WT were similar (**Figure 3A**). However, the *SlHSP70* relative expression in PR3v3 is significantly enhanced compared to that of WT at 1- and 16-h heat treatment (**Figure 3A**). The higher transcript levels seem to be also produced at 8 h (*p* ~ 0.07) but not significant as the variation was high among the replicates. This means the level of the *SlHSP70* transcript was the highest at 1 h and reduced with time and still significantly higher compared to WT until 16 h after heat treatment. The transcript levels of *SlHSFA1a* of all edited lines and WT were not significantly different throughout the treatment (**Figure 3A**).

Further assessment of the transcript levels of *SlCu/ZnSOD* and *SlCAT1* before and during heat treatment also revealed no clear correlation between the transcriptional levels of the genes and heat responses except for the transcript of the *SlCAT1* in the PR3v3 line (**Figure 3B**). The accumulation rates of H_2_O_2_ and ${\text{O}}_{2}^{-}$ under heat stress in the tested plants were also revealed by NBT and DAB staining. The NBT data show similar accumulation rates of H_2_O_2_ among the tested plants. However, the DAB data exhibited a stronger ROS-scavenging activity in the edited lines than in the WT control (**Figure 3C**, **Supplementary Figure S9**).

These results indicated that the *SlHyPRP1* knockout allele-carrying lines exhibited heat tolerance in both tomato varieties [Hongkwang (HK) and 15T01], although the tolerance might not strongly correlate with the transcriptional expression of the heat stress-responsive genes *SlHSP70* and *SlHSFA1a* during stress treatments. The 15T01 KO line PR3v6 that did not show heat tolerance might be because of the extra a.a. at the C-terminal of the protein that negatively affects the heat responses of the line (**Supplementary Figure S2**).

### Drought stress-tolerant performance of domain KO lines

#### The 8CM removal lines showed better germination under osmotic stress

The assessment of osmotic stress responses was conducted following Li and coworkers’ protocol with a minor modification (Li et al., 2016). The seeds of six edited lines (PR1, PR1v1, PR2v2, PR2v3, PR3v1, and PR3v4) from the HK variety were sowed on ½ MS medium with and without 200 mM of mannitol. Twelve days after seed sowing, the seeds of the 8CM removal lines (PR2v2 and PR2v3) germinated better than WT control seeds on the medium containing 200 mM of mannitol in stem and root length; meanwhile, there was a significantly lower germination rate in that of KO lines (PR3v1 and PR3v4) and the PRD removal line (PR1) (**Supplementary Figures S10**, **S11**). Moreover, although the PR1v1 line carrying the 8CM variant allele exhibited significantly lower biomass and stem length than WT, the edited plants showed much more root elongation than WT and the others (**Supplementary Figures S10**, **S11**).

#### The PRD removal line exhibited enhanced drought tolerance at the vegetative growth stage

To assess the edited lines’ ability to tolerate drought conditions, the plants were subjected to water cutoff for 5 days and recovered for 1 week. Only the PR1v1 (PRD removal variant) line was not wilted after drought exposure (**Supplementary Figure S12**). The PR2v3 plants appeared partially wilted. However, no significant variation in the recovery rate and the other growth parameters was observed between the edited lines and WT (**Supplementary Figures S12**, **S13**). The accumulation of ROS was also not different among most of the tested lines, except PR1v1 and PR2v3, with a slight reduction post-treatment (**Supplementary Figure S14**).

### Expression of defense marker genes in *SlHyPRP1*-edited plants

**Figure 4:**
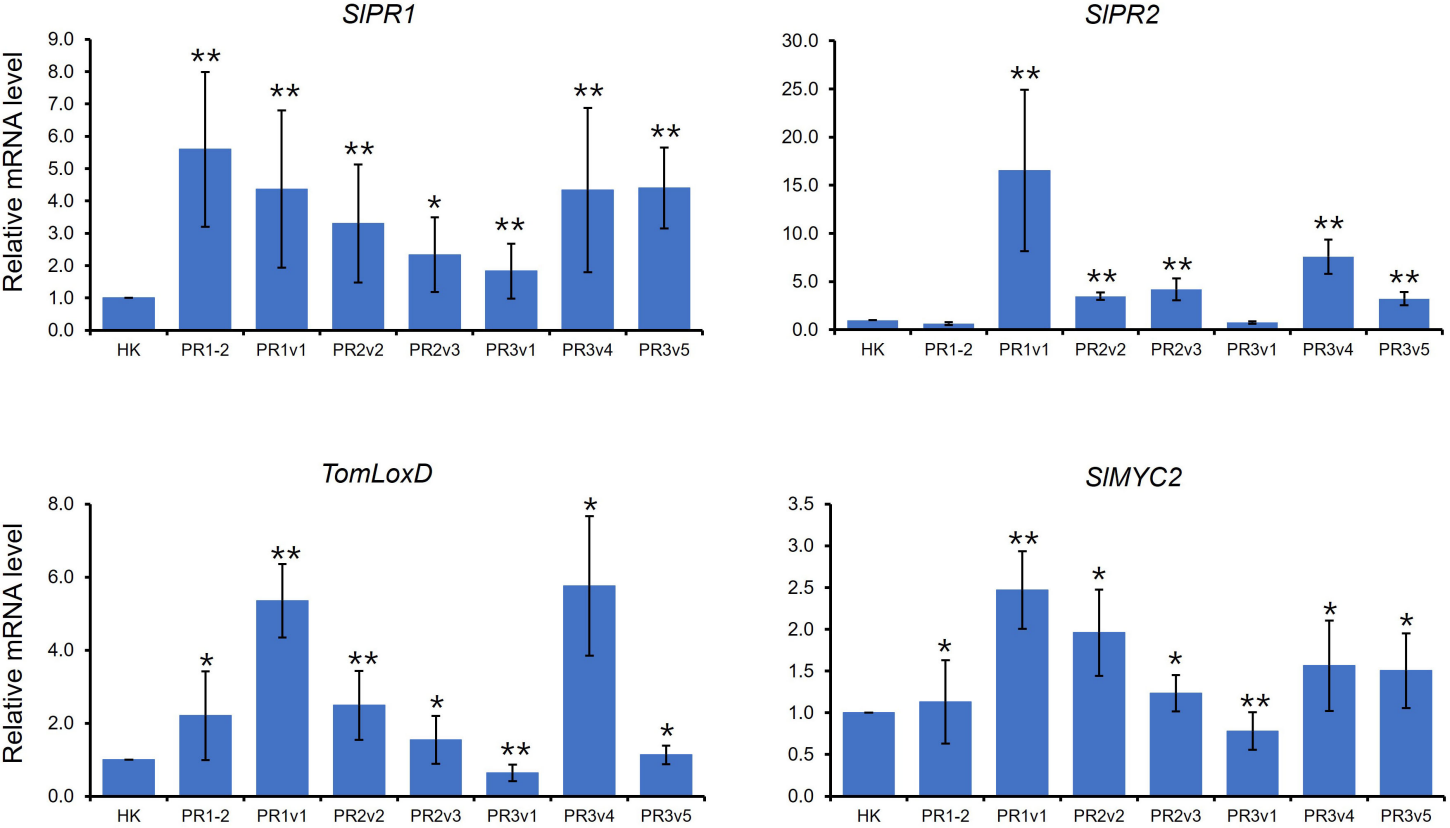
Expression of defense marker genes in HK-based Sl*HyPRP1* edited lines. Relative mRNA expression of SA- and JA-related genes. The genes used for gene expression analysis refer to *SlPR1* (*Solyc09g007010.1*), *SlPR2* (*Solyc01g008620.2*), *TomLoxD* (*Solyc03g122340.2*), and *SlMYC2* (*Solyc08g076930*). Gene expression levels of each gene were normalized with *SlGAPDH* (*Solyc04g009030.2*) and *SlACT* (*Solyc04g011500.3.1*) as an internal control. Gene expression was repeated once, respectively, with similar results. Error bars indicate standard deviation. The *t*-test was conducted for comparison (*p< 0.05, **p< 0.01).

The defense-related genes, *NbPR1a, NbPR2*, and *NbSAR8.2*, were downregulated in *Nicotiana benthamiana (N. benthamiana)* overexpressing pepper *CaHyPRP1* or were upregulated in *N. benthamiana* silencing *NbHyPRP1* (Yeom et al., 2012). Like *NbHyPRP1-*silenced plants, expression levels of salicylic acid (SA) marker genes, *SlPR1* and *SlPR2*, were increased in overall *SlHyPRP1*-edited plants compared to wild-type HK (**Figure 4**). TomLoxD is involved in jasmonic acid (JA) biosynthesis (Hu et al., 2015). AtMYC2, a basic helix-loop-helix transcription factor, plays an important regulatory role in the MYC2 branch of the JA pathway (Dombrecht et al., 2007; Verhage et al., 2011). As shown in **Figure 4**, *TomLoxD* and *SlMYC2* were upregulated in overall *SlHyPRP1*-edited plants, suggesting that both SA- and JA-dependent defense are upregulated in untreated *SlHyPRP1*-edited lines.

### Enhanced resistance to *Pto* DC3000 in the Sl*HyPRP1*-edited HK

**Figure 5:**
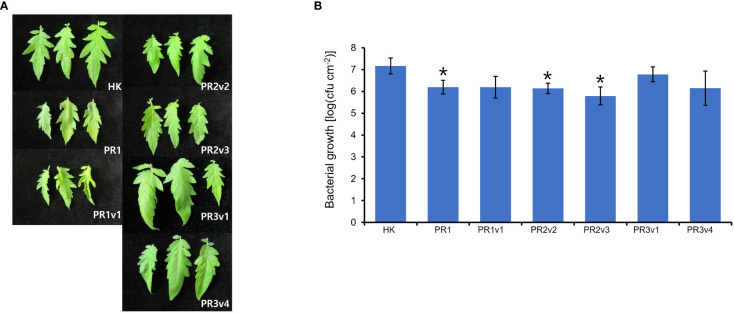
Plant response to *Pseudomonas syringae* pv. *tomato* DC3000 in HK-based *SlHyPRP1* edited lines. **(A)** Disease symptoms of parental HK, PR1, PR1v1-1, PR2v2-1, PR2v3-1, PR3v1-1, and PR3v4-1 dip-inoculated with *Pto* DC3000. Disease symptoms were evaluated at 3 DPI. **(B)** *In planta*, bacterial growth was measured in *SlHyPRP1*-edited plant lines 3 days after inoculation with *Pto* DC3000 at a 1 × 10^8^ cfu/ml density. Values represent averages of cfu/cm^2^ leaf tissue from three replicas, and error bars denote standard deviation. Asterisks indicate that the growth of *Pto* DC3000 was statistically different between HK and PR1-2, PR2v2-1, and PR2v3-1 mutants (*t*-test,*p< 0.01). This experiment was performed twice with similar results.

Silencing *HyPRP1* in cotton enhanced resistance to *Verticillium dahlia*, a hemibiotrophic fungal pathogen (Yang et al., 2018). The increased susceptibility to virulent *Pseudomonas syringae* pv. *tabaci* is observed in *N. benthamiana* overexpressing pepper *CaHyPRP1*, but the disease development was the opposite in *CaHyPRP1-*silenced *N. benthamiana* (Yeom et al., 2012). To test whether the level of resistance to (hemi-) biotrophic virulent bacteria is altered in *SlHyPRP1-*edited lines, we challenged 6-week-old HK, PR1, PR1v1, PR2v, PR3v1, and PR3v4 with *Pto* DC3000 by partial dipping method. Interestingly, the growth of *Pto* DC3000 in PR1, PR2v2, and PR2v3 lines was significantly 10-fold reduced than that in wild type and bacterial growth in PR1v1, PR3v1, and PR3v4 was approximately twofold reduced than that in wild type on average (**Figure 5**). These results suggest that increased SA-pathway defense genes in *SlHyPRP1*-edited plants lead to resistance to the (hemi-) biotrophic pathogen *Pto* DC3000. Therefore, SlHyPRP1 negatively regulates the immune response against the (hemi-) biotrophic pathogen *Pto* DC3000, and the function of this protein is conserved in crops.

### Enhanced susceptibility to the necrotrophic fungal pathogens *Fusarium oxysporum* f. sp. *lycopersici* in the *SlHyPRP1* precisely edited HK and 15T01 lines

**Figure 6:**
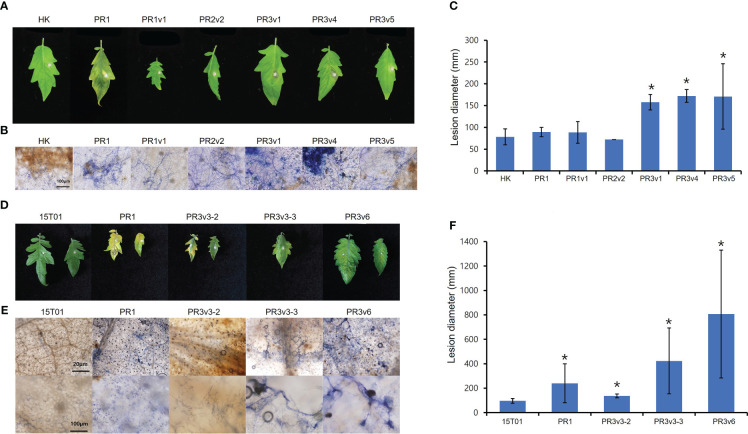
Plant response to *Fusarium oxysporum* f. sp. *lycopersici* in HK- and 15T01-based *SlHyPRP1* edited lines. Disease development was evaluated 6 days after *FOL* inoculation in HK-based **(A–C)** and 15T01-based **(D–F)** *SlHyPRP1* edited lines. **(A, D)** Detached leaves from 6-week-old wild-type plants and *SlHyPRP1* edited mutants grown in a 16-h light/8-h dark long-day photoperiod were inoculated with small plugs of *FOL*. Photographs were taken at 6 DPI. **(B, E)** Trypan blue staining in *FOL*-inoculated *SlHyPRP1* lines. **(C, F)** Lesion size at 6 DPI in *SlHyPRP1* edited lines. The experiment was repeated more than twice with similar results. The statistical analysis was performed using the *t*-test (*p< 0.02).

To identify the role of HyPRP1 in *Fusarium oxysporum* f. sp. *lycopersici* (*FOL*)–plant interaction, the tomato wild-type cultivar HK and 15T01, and *SlHyPRP1-*edited lines (PR1, PR2, and PR3) were plug-inoculated with *FOL*. Six days after plug inoculation with *FOL*, overall *SlHyPRP1*-edited variants displayed increased disease symptoms compared to wild-type HK (**Figure 6A**). Lesion size in the *SlHyPRP1*-edited variants was more than two times larger than in wild-type HK (**Figure 6B**). Trypan blue staining of the inoculated leaves revealed that fungal hyphae were intensively developed in *SlHyPRP1*-edited variants (**Figure 6C**). To test the cultivar-dependent role of HyPRP1 to necrotroph, 15T01 cultivar background *SlHyPRP1*-edited variants (PR1-3, PR3v3-2, PR3v3-3, and PR3v6) were analyzed for the response to *FOL.* Consistent with the results in *SlHyPRP1*-edited HK, severe symptoms, such as enhanced chlorosis, highly extended fungal hyphae, and larger lesion size, were observed in *SlHyPRP1*-edited 15T01 variants (**Figures 6D–F**). Together, these results suggest that *SlHyPRP1*-edited variants increased susceptibility to *FOL* in at least two different tomato cultivars, and SlHyPRP1 plays a positive role in regulating the plant defense against necrotrophic fungal pathogens.

## Discussion

### Edited lines carrying variant alleles expressed heat stress tolerance

The unforeseeable climate changes, such as those generating heat waves throughout the world this summer, have been posing risks to global food production and, thus, the sustainability of agriculture (Shekhawat et al., 2022). Heat stress is becoming a critical target for crop improvement. In this study, we examine the heat stress tolerance of genome-edited lines carrying a different arrangement of SlHyPRP1 domains that were previously shown to tolerate saline treatments in germination and vegetative growth stages (Tran et al., 2020).

The reduced transcription of *SlHyPRP1* during heat treatment may implicate that it plays a negative role in heat stress responses (Li et al., 2016). Therefore, we expect some of the *SlHyPRP1*-edited alleles to exhibit heat tolerance. Interestingly, all the heat-tolerated lines do not carry a functional 8CM motif, indicating that it negatively affects the heat responses of the tomato (**Figures 1**, **2**). The 8CM domain was shown to mediate induced plant cell death in pepper and tobacco (Yeom et al., 2012). As the domain is highly conserved in plants (Li et al., 2016), the enhanced heat tolerance in the 8CM removal and KO lines might be due to reduced sensitivity to cell death. Moreover, it would be interesting to analyze the interaction between the domains and the regulated transcription factors associated with them. However, the prospective work may reach beyond the scope of this study.

HSP and HSFA1 proteins are the master regulator of heat responses (Hahn et al., 2011; Liu et al., 2013) and strictly control each other to activate a cascade of heat-inducible genes (Ohama et al., 2016; Tiwari et al., 2020). We selected *SlHSP70* and *SlHSFA1a* as the marker genes for heat stress response analysis according to previous publications showing the relationships of heat-tolerant *SlMAPK3*-knocked out lines also generated by CRISPR-Cas9 (Yu et al., 2019) and the characterization of functional domains of CaHyPRP1 by Yeom and coworkers in 2012 (Yeom et al., 2012). Furthermore, HSP70 is one of the critical molecular chaperones in plants that help to maintain and restore protein homeostasis, which is necessary for plant survival under heat stress (Hahn et al., 2011). HSFA1a is an essential regulator for heat stress-induced gene expression and thermotolerance (Liu et al., 2013); however, its transcription is not heat stress-inducible and is accompanied by the HS-inducible HSF2 and HSFB1 (Lyck et al., 1997). In Arabidopsis, there is redundancy in functions among several HSFA1 proteins, including HSFA1a, HSFA1b, and HSFA1d (Hahn et al., 2011). Therefore, it is explainable that there was a very weak correlation between the activation of *SlHSP70* and *SlHSFA1a* in the heat-tolerant edited lines. It is also possible that there are significant cross-talks between the SlHyPRP1 domains with heat-inducible proteins from the downstream of the SlHSP70 and SlHSFA1a cascade, such as HSP101/HSFA2, HSP100, HSFBs, DREB2A, and HSC70 (Ohama et al., 2017; Tiwari et al., 2020). Thus, deeper *in silico* analysis to identify heat-related marker genes and assess their transcript levels is required in further works to understand the underlying mechanism behind heat tolerance in the edited lines.

Heat stress often induces the overproduction of ROS. High ROS production in plant tissues can result in oxidative damage, affecting cell activity (Hasanuzzaman et al., 2014). Antioxidant enzymes, including SOD and CAT1, are crucial in ROS detoxification and may participate in thermal stress adaptation. Their strengths positively correlate with plant acquisition of heat tolerance (Tutar et al., 2017; Esposito et al., 2018). Therefore, we also evaluate *Cu/ZnSOD* transcript levels in edited lines of HK cultivar during the heat stress of this experiment but revealed a non-significant difference in all edited lines compared with WT before and after 72 h heat treatment (**Supplementary Figure S6**), possibly due to a very long sampling period. However, shorter data points in 15T01 background also revealed similar data on the transcript levels of the heat stress regulators and the ROS-scavenging genes, indicating the consistency of mechanism relating to HyPRP1-mediated heat responses in the background. Further works can be investigated to reveal the transcription of other ROS-scavenging markers, such as ascorbate peroxidase (APX) or peroxidase (POD) (Ohama et al., 2017; Yu et al., 2019), which might act redundantly in the edited lines.

### Edited lines carrying variant alleles expressed drought stress tolerance

The drought stress response is another factor that negatively impacts crop yield (Farooq et al., 2009; Farooqi et al., 2020; Seleiman et al., 2021). Osmosis stress is a good marker for drought and salinity. Previously, we demonstrated that some of the *SlHyPRP1*-edited lines were salt-tolerant. In this study, the 8CM-deleted *SlHyPRP1* allele-carrying plants grew better than WT in the osmosis conditions during the germination stage. In contrast, the PRD-deleted lines negatively affected the edited plants, indicating that either the polypeptides containing only the PRD domain and the signal peptide positively affect the osmotic tolerance of the plants, or the 8CM motif alone negatively affects the process. The PRD alone, when translocated to the plasma membrane zone, might positively enhance the osmosis stress responses. However, the data revealed from the drought treatment indicates different impacts of the edited alleles in the vegetative growth stage. Our data are quite different from the report of the RNAi-mediated *SlHyPRP1* silencing approach (Li et al., 2016), possibly because of either the incomplete silencing of *HyPRP1* or multiplicative silencing of homologous genes in RNAi lines. More work should be performed to understand the specific mechanism in regulating osmosis and drought responses by the SlHyPRP1 domains.

### Edited lines carrying variant alleles oppositely regulated resistance to biotrophic and necrotrophic pathogens

*SlHyPRP1*-edited plants showed enhanced stress tolerance against salinity, high temperature, and osmosis/drought in previous and current studies. To identify the role of SlHyPRP1 in the plant resistance to pathogens, we applied two types of plant pathogens into *SlHyPRP1*-edited plants: *Pto* DC3000, a hemibiotrophic pathogenic bacterium, and *FOL*, a necrotrophic pathogenic fungus. First, bacterial growth levels of *Pto* DC3000 were lower in *SlHyPRP1*-edited plants than in wild-type plants (**Figure 5**). Second, two different cultivar background *SlHyPRP1*-edited plants are more susceptible to a necrotrophic fungal pathogen *FOL* than wild-type plants (**Figure 6**). Third, the basal expression levels of the SA-related and JA-related defense marker genes were upregulated in overall *SlHyPRP1*-edited plants (**Figure 4**).

The HyPRP family from different plant species has been known to respond to abiotic stresses, plant pathogens, and plant hormones (Huang et al., 2011; Yeom et al., 2012; Neto et al., 2013; Tan et al., 2013; Li et al., 2016; Yang et al., 2018; Banday et al., 2022). Plant resistance against biotrophic pathogens, which feed on living plant cells, is mediated by the SA signaling pathway, while resistance to necrotrophic pathogens, which kill host cells and utilize nutrients for their growth, is dependent on the JA and/or the ethylene signaling pathway (Glazebrook, 2005). The expression of *pGbHyPRP1-GUS* was increased in response to JA and ethylene but decreased by applying SA (Yang et al., 2018). Downregulation of cotton *HyPRP1* revealed increased resistance against a hemibiotrophic fungal pathogen, *V. dahlia*, and overexpression of cotton *HyPRP1* in Arabidopsis was more susceptible to it (Yang et al., 2018). The silencing of *HyPRP1* in *N. benthamiana* resulted in increased resistance to *P. syringae* pv. *tabacci* and upregulated expression of SA-related defense marker genes, *NbPR1a*, *NbPR2*, and *NbSAR8.2* (Yeom et al., 2012). Consistent with these reports, CRISPR-induced mutation in *SlHyPRP1* elevated SA marker genes and conferred resistance to *Pto* DC3000 (**Figure 5**).

Oppositely, some PRPs and cell-wall proteins are involved in resistance to necrotrophic fungal pathogens. Recombinant protein of JsPRP1 (*Juglans sigillata* Dode PRP) conferred *in vitro* antifungal activity to *Colletotrichum gloeosporioides*, and *JsPRP1* transgenic tobacco plants increased tolerance to *C. gloeosporioides* (Liu et al., 2018). In addition, the recombinant protein of EARLI1, one of the Arabidopsis HyPRP, inhibited the growth of fungal pathogens, necrotroph *B. cinerea* and *F. oxysporum* (Li et al., 2012). In Arabidopsis, overexpression of the extracellular plant proteins, polygalacturonase-inhibiting proteins (PGIP1 and PGIP2) that counteract with cell wall-degrading enzyme polygalacturonase secreted from fungi, led to enhanced resistance to *B. cinerea* (Ferrari et al., 2003). These results and our findings demonstrate that some HyPRP positively regulates plant response to a necrotrophic fungal pathogen (**Figure 6**). However, the role of SlHyPRP1 in interaction with necrotrophic fungal pathogen is unclear. Measurement of JA or expression of JA-responsive genes during necrotrophic fungal pathogen infection in *SlHyPRP1*-edited alleles will be of particular interest.

*SlHyPRP1*-edited plants revealed an opposite immune response against a hemibiotrophic bacterial pathogen and a necrotrophic fungal pathogen (**Figures 5**, **6**). Mutations in genes such as *BOTRYTIS-INDUCED KINASE1* (*BIK1*), *Botrytis Susceptible 1 (BOS1)*, and *SUPPRESSOR OF rps4-RLD1* (*SRFR1*) had an opposite phenotype against biotrophic and necrotrophic pathogens (Mengiste et al., 2003; Veronese et al., 2005; Son et al., 2021). Additionally, SA accumulation was increased in Arabidopsis *atbik1* mutant (Veronese et al., 2005). The SA marker genes were upregulated in *atsrfr1 and slsrfr1* mutants similar to *SlHyPRP1*-edited plants (Nguyen et al., 2016; Son et al., 2021). Therefore, enhanced resistance in *SlHyPRP1*-edited plants to hemibiotrophic *Pto* DC3000 likely results from the upregulation of SA pathways, which, in turn, suppress JA pathways to confer susceptibility to necrotrophic *FOL*. However, the function of SlHyPRP1 in SA-mediated defense is not validated. Accordingly, investigation of endogenous level of SA in *SlHyPRP1*-edited lines is further required. Consequently, precise GE mediated by the CRISPR-Cas system would have important implications for crop breeding and genetic engineering efforts to improve tolerance to abiotic stresses and pathogens.

## Conclusions

CRISPR-Cas-based targeted mutagenesis is a revolutionary crop improvement technique that could help meet food production demand. The CRISPR-Cas technology is being engineered with negative stress response regulators to obtain improved crops capable of dealing with changes in growing conditions. The CRISPR-Cas9 was successfully applied in our previous study to obtain precisely edited alleles and their variants of the target gene, *HyPRP1*, a negative regulator of multi-stress responses. In this study, multi-stress-tolerant tomato plants have been successfully demonstrated. It is important to note that careful design in multi-domain gene editing is required to obtain the best result of CRISPR-driven breeding. New strategies considering domain activities after GE rather than producing a perfect loss of gene function could provide a broad spectrum of edited traits (**Supplementary Table S2**).

Overall, this study demonstrated that the CRISPR-Cas9 system is a powerful technology to edit multiple target sites in a single gene and generate precise GE in both the tomato genetic backgrounds (HK and 15T01), resulting in multi-stress tolerance. This can be easily extended to a broader range of crops for creating expected multiple traits pyramiding with elite alleles that function for stress-tolerant, high-quality, and high-yield crops.

## Materials and methods

### Heat test tolerance assay

The heat test protocol followed that of Yu et al. (2019) with minor modifications (Yu et al., 2019). Briefly, seedlings of similar size were selected to evaluate the heat stress tolerance of edited lines compared with WT and transformed WT-like plants. Six-week-old seedlings of Genome Editing Generation 3 (GE3)- and GE4-edited lines with WT and WT-like HK were tested at high temperatures for 9 days (3 days at 42°C and then 6 days at 45°C) in replicate #1; 6 days at 45°C in replicate #2; and 6 days (3 days at 42°C and then 3 days at 45°C) in replicate #3. Five-week-old seedlings of GE1- and GE2-edited lines with WT of 15T01 cultivar were placed at 42°C/42°C (day/night) in the growth chamber for 16 h. Young leaves from six stress-treated plants were collected at each time point (0, 1, 8, and 16 h for the 15T01 cultivar; 0, 24, 48, and 72 h for the HK cultivar) after heat treatment and flash-frozen in liquid nitrogen. All the experiments were conducted under 16-h light/8-h dark photoperiods, and then plants were moved to 25°C (16-h light/8-h dark) conditions for recovery in 1 month. Data were collected in triplicate during the testing and recovery stages after heat treatment.

### Drought test tolerance assay

#### Drought stress tolerance in the germination stage

Homozygous edited lines and WT HK and WT-like seeds were sterilized and grown on ½ MSO medium for 4 days [3 days in the dark and 1 day in the light (16-h light and 8-h dark photoperiods)]. The germinated seeds on the fourth day were transferred into ½ MSO medium supplemented with different concentrations of mannitol consisting of 0 and 200 mM, pH 5.7. The fresh weight, stem length, and root length data were recorded on the 12th day after being subjected to mannitol.

### Drought stress tolerance in the growth stage

Drought stress treatment was carried out according to Wang et al. (2017a) with minor modifications. Briefly, the seedlings of the same size were selected to evaluate the drought stress tolerance of edited lines compared with WT. The 6-week-old plants did not receive water for five consecutive days in the culture room under the same conditions and then were re-watered and photographed for a 1-week recovery. Young leaves from stress-treated plants were collected before and 5 days after removing water for RNA extraction. Retained water in soil, leaves, and plants was recorded on the fifth day after watering was stopped. Ten leaves of each plant, all the plants with roots, and soil were collected and recorded. The initial fresh weights were kept in a dry oven (60°C) for 24 h. Three biological replicates were conducted, and data were analyzed and collected during testing and when water supply was discontinued for drought stress treatment.

### Histochemical detection of singlet oxygen O^2-^ radical and hydroperoxide H_2_O_2_

Singlet oxygen O^2−^ radical and H_2_O_2_ levels were revealed by NBT and DAB stainings, according to Ijaz et al. (2017). Briefly, fully expanded leaves on the second compound leaves were collected at 0 h before stress treatment and 72 h after heat treatment for the HK cultivar, 16 h after heat treatment for the 15T01 cultivar, and 5 days after drought treatment. The leaves were soaked in 1 mg/L NBT [using 50 mM sodium phosphate buffer (pH 7.5); the mixture of 16 ml of 1 M sodium phosphate monobasic (NaH_2_PO_4_) and 84 ml of 1 M sodium phosphate dibasic (Na_2_HPO_4_) solutions and 2 L volume was made using distilled water, and then adjusted to pH 7.5] and 1 mg/L DAB (pH 3.8) solution. After staining, the leaves were put in 95% ethanol and changed to ethanol 95% 1 and 2 days later until chlorophyll was removed entirely. Photographs were taken any time after that, and fresh 95% ethanol could be used during the photo process to avoid dried samples.

### Bacterial and fungal pathogenesis assay

The growth assay using *Pseudomonas syringae* pv. *tomato* (*Pto*) DC3000 was performed as previously described (Son et al., 2021). *Pto* DC3000 containing the pML123 empty vector was grown in selective *Pseudomonas* agar plates at 28°C. Bacterial cells were suspended in water with 0.01% silwet at 1 × 10^8^ CFU/ml. The leaves in 6-week-old tomato were dipped into a suspension of bacteria for 1 min and then placed in a plastic bag with high humidity to promote the growth of the bacteria on the leaf surface. Two leaf discs with a cork borer (No. 5) were collected and ground in 400 μl of 10 mM MgCl_2_, and suspensions were plated in serial dilutions on a selective *Pseudomonas* agar media (four replicas) at 3 days after dip inoculation.

*FOL* was plug-inoculated on the detached leaves as performed (Son et al., 2021). *FOL* grown on potato dextrose (#254920, BD Difco, Franklin Lakes, USA) were made into little pieces with a cork borer (No. 1). Small plugs were put on detached tomato leaves. Tomato leaves placed on black trays were covered with plastic wrap and incubated at 25°C (16 h light/8 h dark) for 6 days.

### Trypan blue staining

Trypan blue staining was conducted as previously reported (Fernández-Bautista et al., 2016; Son et al., 2021). Tomato tissues infected with *FOL* were cut and submerged into trypan blue solution (1:1:1 = lactic acid:phenol:glycerol with a final concentration of 10 mg/ml of trypan blue). Then, they were incubated with gentle shaking for 1 h. Trypan blue solution and chlorophyll were removed gradually with 99%, 70%, and 50% ethanol.

### RNA isolation, RT-PCR and qRT-PCR procedure, and data analysis

#### In abiotic treatment assays

Sample preparation and qRT-PCRs followed MIQE guidelines (Bustin et al., 2009). Total RNA was extracted with a miniprep kit (cat. no. 74104, Qiagen, USA). Total RNA concentration was checked using NanoDrop 2000 (Thermo Scientific, Waltham, MA, USA). First, cDNA strands were synthesized from 1 µg of total RNA to reverse transcription using the QuantiTect Reverse Transcription Kit and protocol (cat. no. 205311, Qiagen) and then cDNA was used for RT-PCR and qRT-PCR with intercalating dyes (KAPA SYBR FAST Universal, cat. No. KK4601, Sigma, USA) to detect products. Forward and reverse primers used for RT-PCR were designed to flank the signal peptide (SP) and 3’UTR: 5’-CAGTCgaagacaaAATGATGGAGTTCTCTAAGATAACTT CA CTTCTT-3’ and 5’-AACAATTCCACAAAGCCAAA-3’, respectively. At least two pairs of all primers (**Supplementary Table S3**) were tested for each target gene/cDNA to evaluate their efficiencies, and primer pairs with ~100% efficiency were ultimately used for qRT-PCR. A similar assessment was also applied to the internal genes to normalize the amplicon levels. Thermocycling for qRT-PCR was conducted with the Illumina Eco Real-Time PCR System (Illumina, USA) as follows: (1) 95°C for 3 min, followed by 40 cycles from step 2 to step 4 at (2) 95°C for 8 s, (3) 60°C for the 30 s, (4) 95°C for 15 s, (5) 55°C for 15 s, and (6) 95°C for 15 s. Analyses of amplicon levels were performed using the delta-delta Cq method (Livak and Schmittgen, 2001) with the internal gene/transcript of SlPDS. They were plotted using GraphPad Prism 9.0 or Excel software (Microsoft, USA), and SlPDS was used for normalization. The analysis was conducted in three biological replicates for assessing transcription of tomato oxidative-related gene superoxide dismutase *SlCu/ZnSOD* (Solyc01g067740.2.1), catalase *SlCAT1* (Solyc12g094620.1.1), and the expression of heat shock protein related to gene *SlHSP70* (Solyc11g020040.1) and transcription factor gene *SlHSFA1a* (Solyc08g005170). All qRT-PCR primer sequences are listed in **Supplementary Table S3**.

#### Defense-related gene analysis

Total RNA was isolated using RiboEx (#301-001, GeneAll, Seoul, South Korea) following the manufacturer’s instructions. The genomic DNA among 2 μg of total RNA was eliminated with a TURBO DNA-free kit (#AM1907, Invitrogen, Carlsbad, USA). ReverTra Ace-α-™ cDNA synthesis kit (FSK-101F, Toyobo, Osaka, Japan) was used for the synthesis of 1st cDNA following the company’s instructions with reported primers (Mira et al., 2016; Wang et al., 2017b) (**Supplementary Table S3**). Individual gene expression was normalized by the housekeeping genes *SlACT* and *SlGAPDH*. The PCR reactions were carried out on the CFX384 system (BioRad, Hercules, USA) using the IQ SYBR Green (#BR1708880, BioRad, Hercules, USA). The thermocycling program was 95°C for 2 min, 40 cycles (95°C for 5 s), and 60°C for 15 s.

### Statistical analysis

Heat and drought test tolerant data were recorded from 6 to 18 biological replicates of three technical replicates, and the qRT-PCR data were analyzed and plotted using GraphPad Prism 9.0 software. The multiple comparisons of the relative expression levels were performed using an uncorrected Fisher’s LSD test with a threshold of *p*< 0.05.

### Agronomical trait assessment

Morphology, plant height, and stem diameter growth in stress tolerance tests were compared to WT and WT-like.

## Data availability statement

The original contributions presented in the study are included in the article/**Supplementary Material**. Further inquiries can be directed to the corresponding authors.

## Author contributions

TV, SK, and J-YK conceived and designed the research. MT, GS, YJS, NN, SP, TT, JK, YWS, SD, DP, and JL conducted experiments. MT, GS, YS, K-HS, SK, TV, and J-YK analyzed data. MT, GS, YJS, and TV wrote the manuscript. TV, SK, and J-YK finalized the manuscript. All authors contributed to the article and approved the submitted version.
