# Supplementary material for: CRISPR-Cas9-based precise engineering of SlHyPRP1 protein towards multi-stress tolerance in tomato
Source: Front Plant Sci. 2023 May 15;14:1186932. doi: 10.3389/fpls.2023.1186932 (PMC10225705; doi:10.3389/fpls.2023.1186932)
Supplement: Supplementary file 1 [file DataSheet_1.docx]

**SUPPLEMENTAL INFORMATION**

**CRISPR-Cas9-based precise engineering of SlHyPRP1 protein towards multi-stress tolerance in tomato**

Mil Thi Tran^1,2,3†^, Geon Hui Son^1†^, Young Jong Song^1†^, Ngan Thi Nguyen^1^, Seonyeong Park^1^, Thanh Vu Thach^1^, Jihae Kim^1^, Yeon Woo Sung^1^, Swati Das^1^, Dibyajyoti Pramanik^1^, Jinsu Lee^1^, Ki-Ho Son^3^, Sang Hee Kim^1,4*^, Tien Van Vu^1,5,*^, Jae-Yean Kim^1,4,6,*^

^1^Division of Applied Life Science (BK21 Four Program), Plant Molecular Biology and Biotechnology Research Center, Gyeongsang National University, Jinju 660-701, Republic of Korea.

^2^Crop Science and Rural Development Division, College of Agriculture, Bac Lieu University, Bac Lieu 97000, Vietnam.

^3^Division of Horticultural Science, Gyeongsang National University, Jinju 52725, Korea

^4^Division of Life Science, Gyeongsang National University, 501 Jinju-daero, Jinju 52828, Republic of Korea.

^5^National Key Laboratory for Plant Cell Biotechnology, Agricultural Genetics Institute, Km 02, Pham Van Dong Road, Co Nhue 1, Bac Tu Liem, Hanoi 11917, Vietnam.

^6^Nulla Bio Inc 501 Jinju-daero, Jinju 52828, Korea.

^†^These authors share first authorship: Mil Thi Tran, Geon Hui Son, Young Jong Song.

^*^Correspondence: Sang Hee Kim: sangheekim@gnu.ac.kr (ORCID: 0000-0003-1831-4633); Tien Van Vu: tienvu.agi@gmail.com (ORCID: 0000-0002-6369-7664); Jae-Yean Kim: +82-(0)55-772-1361, [kimjy@gnu.ac.kr](mailto:kimjy@gnu.ac.kr) (ORCID: 0000-0002-1180-6232).

**Keywords:** CRISPR-Cas9, HyPRP1, abiotic stress, biotic stress, heat stress tolerance, multi-stress tolerance.

**Running title: *HyPRP1*-edited tomatoes confer multi-stress tolerance.**

| **Supplemental item** | **Page** |
| --- | --- |
| Figure S1. System construction and expected outcomes of the approach. | 3 |
| Figure S2. Amino acid sequence alignment of WT and edited lines from (a) HK variety, (b) 15T01 variety. | 4 |
| Figure S3. PCR products of the edited alleles using gDNAs as templates (HK). | 5 |
| Figure S4. PCR products of the edited allele using gDNAs as templates (15T01). | 6 |
| Figure S5. Heat stress tolerance exhibited by the *SlHyPRP1* precisely edited HK lines. | 7 |
| Figure S6. The relative transcript levels of *SlCu/ZnSOD* of the heat-treated HK lines were quantified by qRT-PCRs. | 8 |
| Figure S7. Effects of the HK-based *SlHyPRP1* edited lines on ROS production (NBT and DAB staining) under heat stress. | 9 |
| Figure S8. Heat stress tolerance exhibited by the edited 15T01 lines. | 10 |
| Figure S9. Effects of the 15T01-based *SlHyPRP1* edited lines on ROS production (NBT and DAB staining) under heat stress. | 11 |
| Figure S10. Performance of edited lines compared to the WT and WT-like at 0 and 200 mM Mannitol. | 12 |
| Figure S11. The biomass accumulation of edited lines, WT, and WT-like at 0 and 200 mM Mannitol. | 13-14 |
| Figure S12. Performance of the edited lines compared to the WT and WT-like plants (HK) after five days under drought stress. | 15 |
| Figure S13. Water content in leaves, plants, and soil and leaf surface area of *SlHyPRP1* edited lines (HK) under drought stress. | 16 |
| Figure S14. Effects of the HK-based *SlHyPRP1* edited lines on ROS production (NBT and DAB staining) under drought stress. | 17 |
| Table S1. Detailed data of the obtained alleles for heat and drought stress treatment | 18 |
| Table S2. Edited allele types showed multi-stress tolerance | 19 |
| Table S3. Primer sequences used in this study | 20-21 |


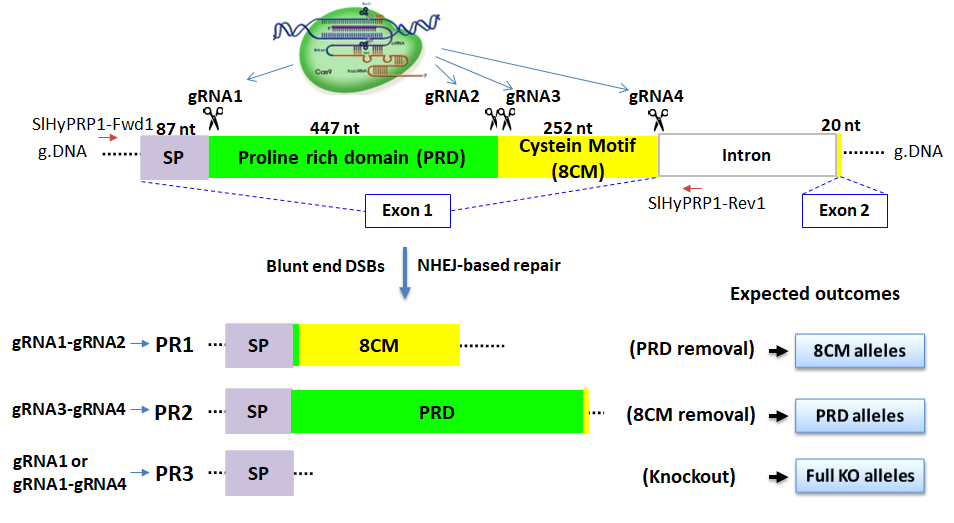


**Figure S1. System construction and expected outcomes of the approach.** A multiplexed CRISPR/Cas9 editing system was used with four guide RNAs (gRNA1, gRNA2, gRNA3, and gRNA4) or in pairs (gRNA1 and gRNA2; gRNA3 and gRNA4) for the targeted double-stranded break (DSB) formation and religation by NHEJ, thereby potentially producing expected products (PR1, PR2, and PR3). The peptide lengths of each part have been denoted above its representative block (nt, nucleotides). SP=signal peptide. SlHyPRP1-Fwd1 and SlHyPRP1-Rev1 were the primer pair for PCR.


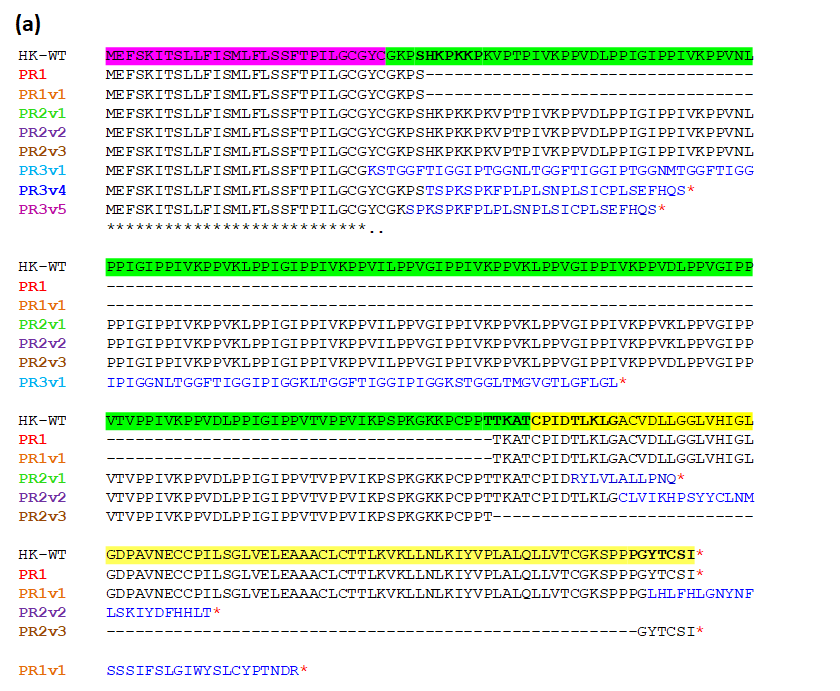


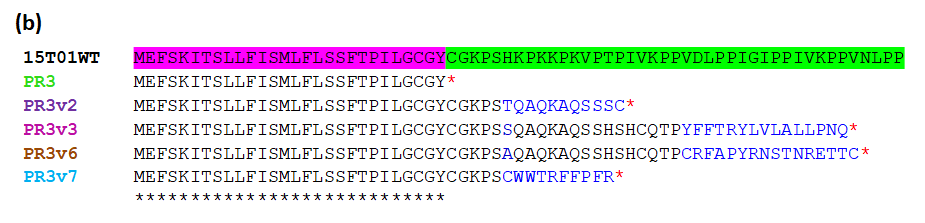


**Figure S2. Amino acid sequence alignment of WT and edited lines from (a) HK variety, (b) 15T01 variety.** PR1: the precise PRD removal events, PR1v1: PRD removal events; PR2v1, PR2v2, and PR2v3: precise 8CM removal variants; and PR3: PRD-8CM removal event, and PR3v1, PR3v3, PR3v4, PR3v5, PR3v6, and PR3v7): PRD-8CM removal variants. Pink highlight: Signal peptide domain, green highlight: Proline-rich domain, yellow highlight: 8-Cysteine Motif domain, blue font: extra sequences, red star: stop codon.


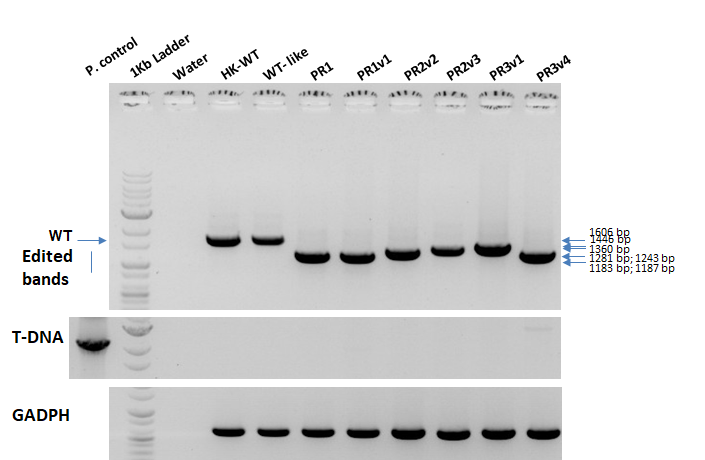


**Figure S3.**  **PCR products of the edited alleles using gDNAs as templates (HK)**. Expected bands of the edited lines: HK-WT: HK wild-type,1606 bp; WT-like: 1606 bp, PR1: 1183 bp; PR1v1: 1187 bp; PR2v2: 1281 bp; PR2v3: 1360 bp; PR3v1: 1446 bp, PR3v4: 1243 bp.


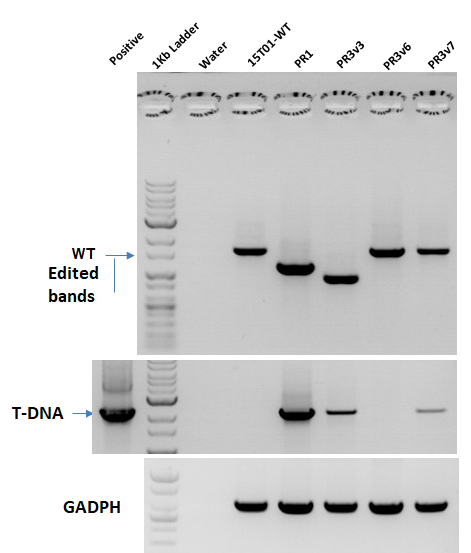


**Figure S4.**  **PCR products of the edited allele using gDNAs as templates (15T01)**. Expected bands of the edited lines: WT (g.DNA): 1606 bp; PR1: 1183 bp; PR3v3: 1543 bp; PR3v6: 1601 bp; PR3v7: 1605 bp.


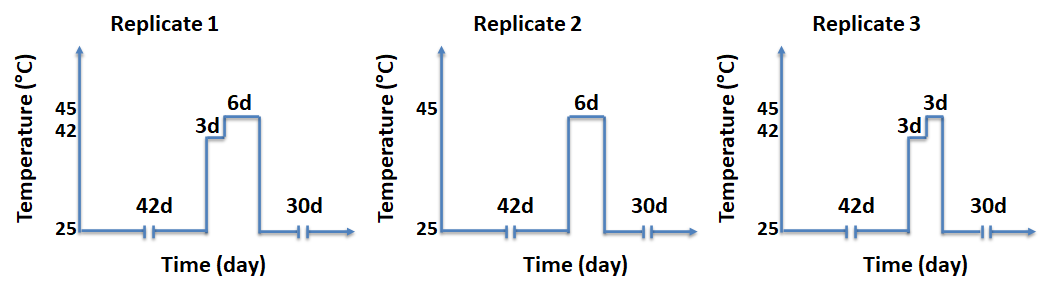


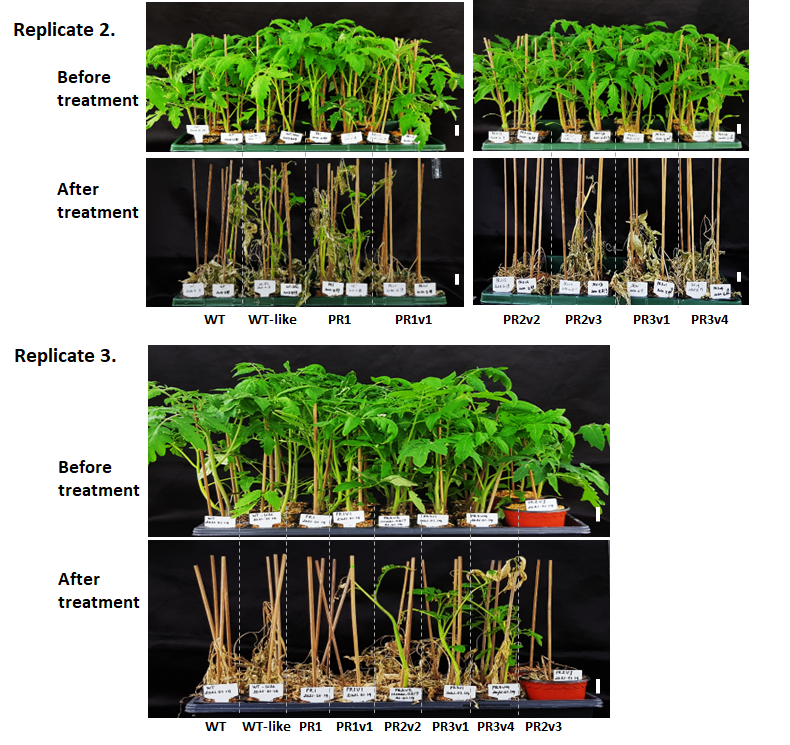


**Figure S5. Heat stress tolerance exhibited by the *SlHyPRP1* precisely edited HK lines.** Plants before treatment and at 30 days post-recovery. Six-week-old seedlings were subjected to high-temperature conditions for nine days (3 days at 42^o^C, and then six days at 45^o^C) in the case of replicate 1, and 6 days at 45^o^C in the case of replicate two under 16h-light/8h-dark in the growth chamber and then moved to 25^o^C (16h-light/8h-dark) in culture room for recovery. Photographs were taken at one-month post-recovery. The name of plants/lines are denoted at the bottom of each panel. Scale bars: 2 cm. WT: wild-type HK cultivar; WT-like: segregated WT siblings of the edited plants; PR1: precise edited event PR1; PR1v1: PR1 variant; PR2v2: variant 2 of precise edited event PR2; PR2v3: variant 3 of precise edited event PR2; PR3v1: variant 1 of precise edited event PR3; PR3v4: variant 4 of precise edited event PR3.


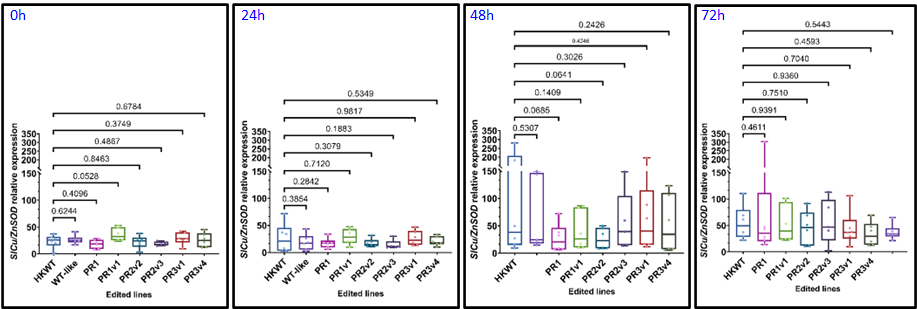


**Figure S6. The relative transcript levels of *SlCu/ZnSOD* of the heat-treated HK lines were quantified by qRT-PCRs**. Leaf tissues of the plants were collected at 0, 24, 48, and 72 h of heat treatment. Total RNAs were then isolated, reversely transcribed into cDNA, and used for qPCRs. The qRT-PCR process was conducted following the MIQE guidelines. The qRT-PCR data were analyzed and plotted using Graphpad Prism 9.0. Data were collected from six biological replicates. The multiple comparisons of the relative expression levels were performed using One-way ANOVA uncorrected Fisher’s LSD test (P<0.05). The p-value is shown on the top of the compared boxes. WT: wild-type HK cultivar; WT-like: segregated WT siblings of the edited plants; PR1: precise edited event PR1; PR1v1: PR1 variant; PR2v2: variant 2 of precise edited event PR2; PR2v3: variant 3 of precise edited event PR2; PR3v1: variant 1 of precise edited event PR3; PR3v4: variant 4 of precise edited event PR3.


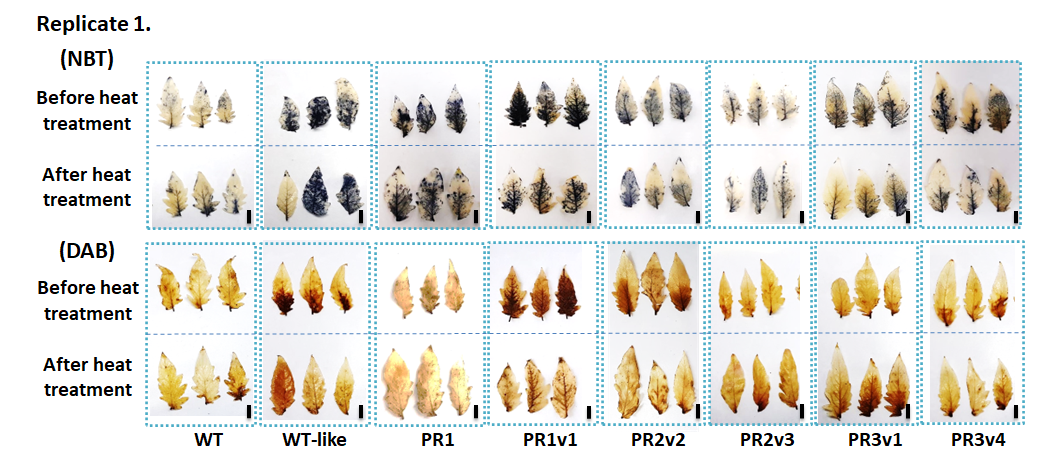

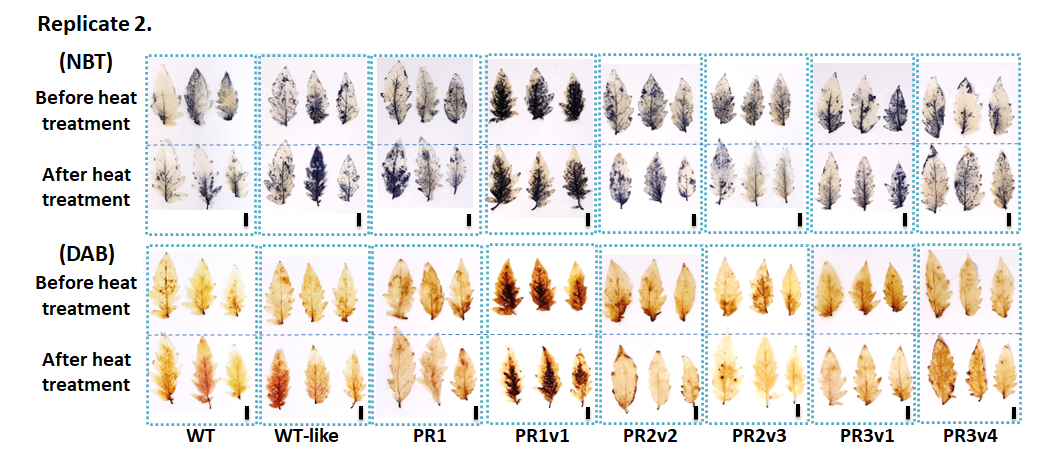


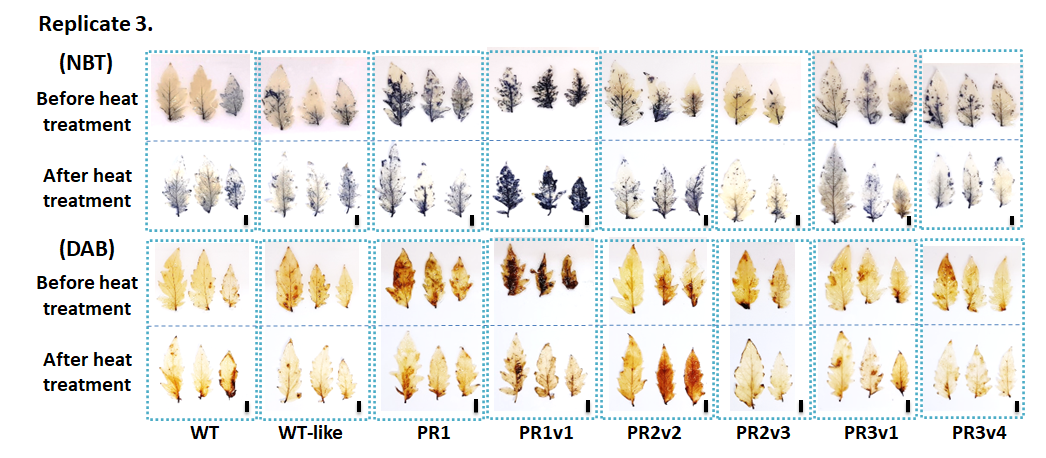


**Figure S7.** **Effects of the HK-based *SlHyPRP1* edited lines on ROS production (NBT and DAB staining) under heat stress.** Leaves of the heat-treated plants were collected before and after 72 h of the heat treatment and stained by the ROS-specific dyes. The top panel of each staining type represents leaves grown at 25°C before heat treatment, and the bottom panel represents leaves of the same plants treated in the heat for 72 h. WT: HK wild-type; WT-like: segregated WT siblings of the edited plants; PR1, PR1v1; PR2v2; PR2v3; PR3v1 and PR3v4: edited lines. Scale bars: 1 cm.


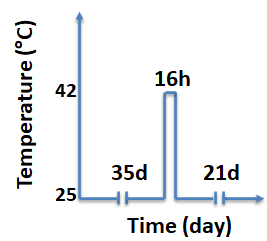


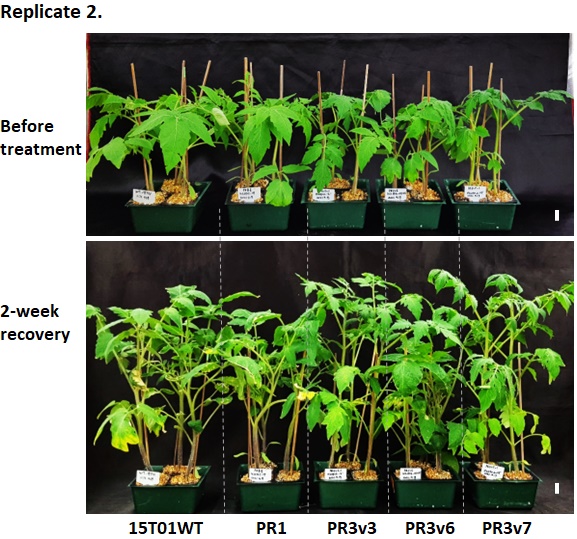


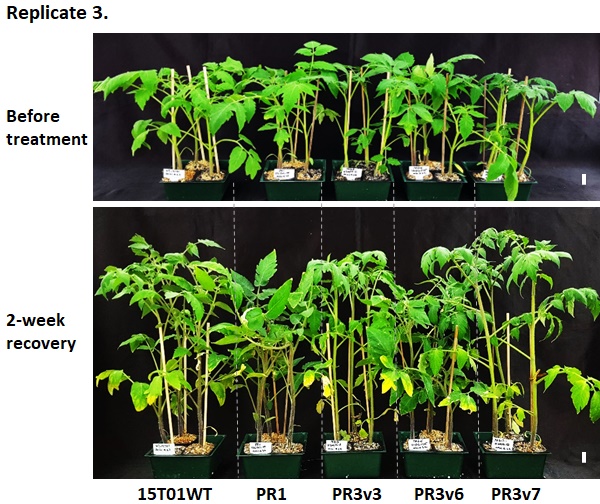


**Figure S8. Heat stress tolerance exhibited by the edited 15T01 lines.** Plants before treatment and at two weeks post-recovery. Five-week-old seedlings were subjected to 42^o^C (16h-light/8h-dark) chamber for 16 hours and then moved to 25^o^C (16h-light/8h-dark) in the culture room. Photographs were taken before treatment and at two weeks post-recovery. The name of plants/lines are denoted at the bottom of each panel. The horizontal lines indicate plants from the same, edited lines. 15T01WT: wild-type 15T01 cultivar, PR1: precise edited event PR1, PR3v3: variant 3 of precise edited event PR3, PR3v6: variant 6 of precise edited event PR3, PR3v7: variant 6 of precise edited event PR3. Scale bars: 2 cm.


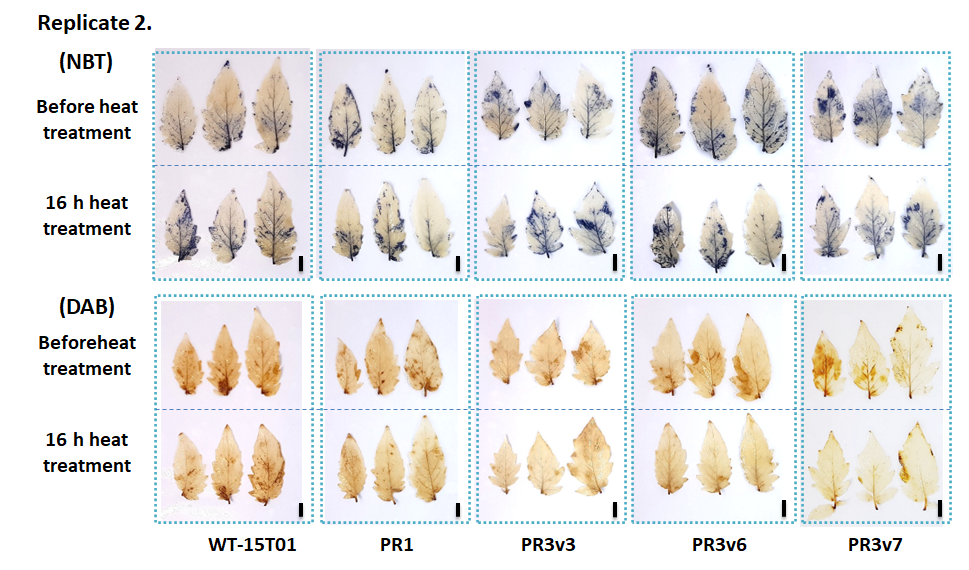


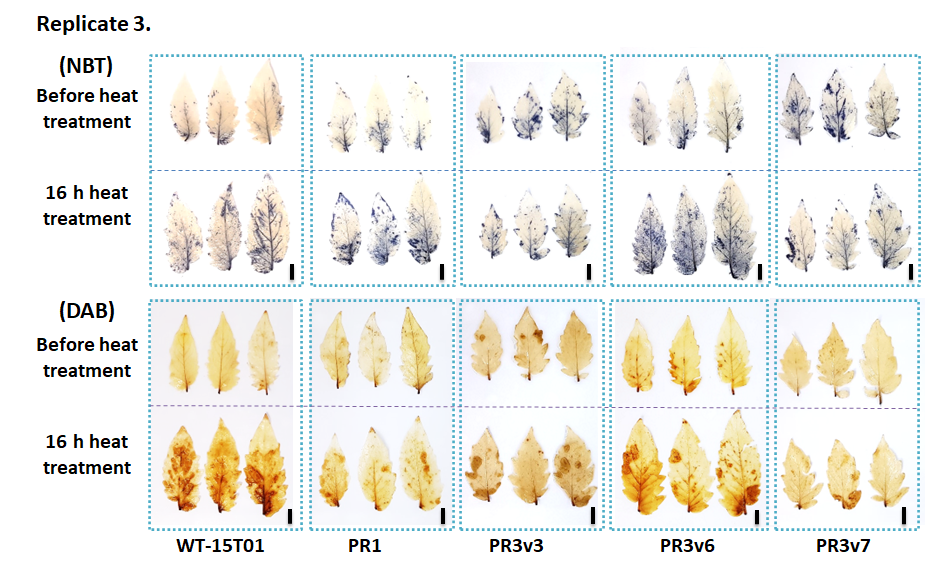


**Figure S9.** **Effects of the 15T01-based *SlHyPRP1* edited lines on ROS production (NBT and DAB staining) under heat stress.** Leaves of the heat-treated plants were collected before and after 16h of the heat treatment and stained by the ROS-specific dyes. The top panel of each staining type represents leaves grown at 25°C before heat treatment, and the bottom panel represents leaves of the same plants treated at 42°C for 16h. 15T01WT: wild-type 15T01 cultivar, PR1: precise edited event PR1, PR3v3: variant 3 of precise edited event PR3, PR3v6: variant 6 of precise edited event PR3, PR3v7: variant 7 of precise edited event PR3. Scale bars: 1 cm.


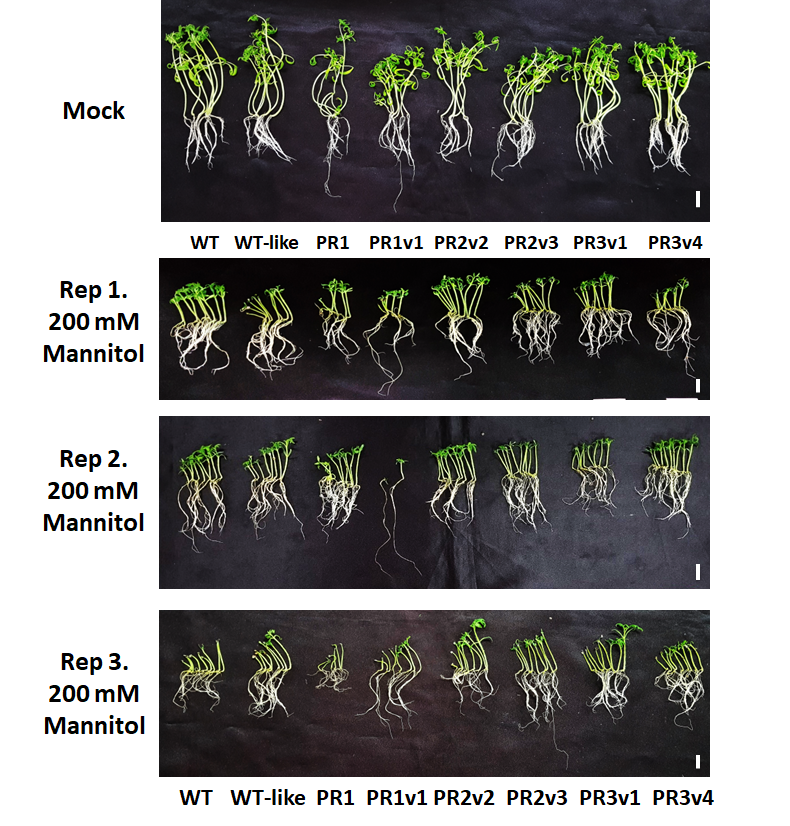


**Figure S10. Performance of edited lines compared to the WT and WT-like at 0 and 200 mM Mannitol.** The data were analyzed and plotted using Graphpad Prism 9.0. Data were collected from 10 to 24 biological replicates. The multiple comparisons of the relative expression levels were performed using One-way ANOVA uncorrected Fisher’s LSD test (P<0.05). The p-value is shown on the top of the compared boxes. WT: wild-type HK; WT-like: segregated WT siblings of the edited plants; PR1: precise edited event PR1; PR1v1: PR1 variant; PR2v2: variant 2 of precise edited event PR2; PR2v3: variant 3 of precise edited event PR2; PR3v1: variant 1 of precise edited event PR3; PR3v4: variant 4 of precise edited event PR3.


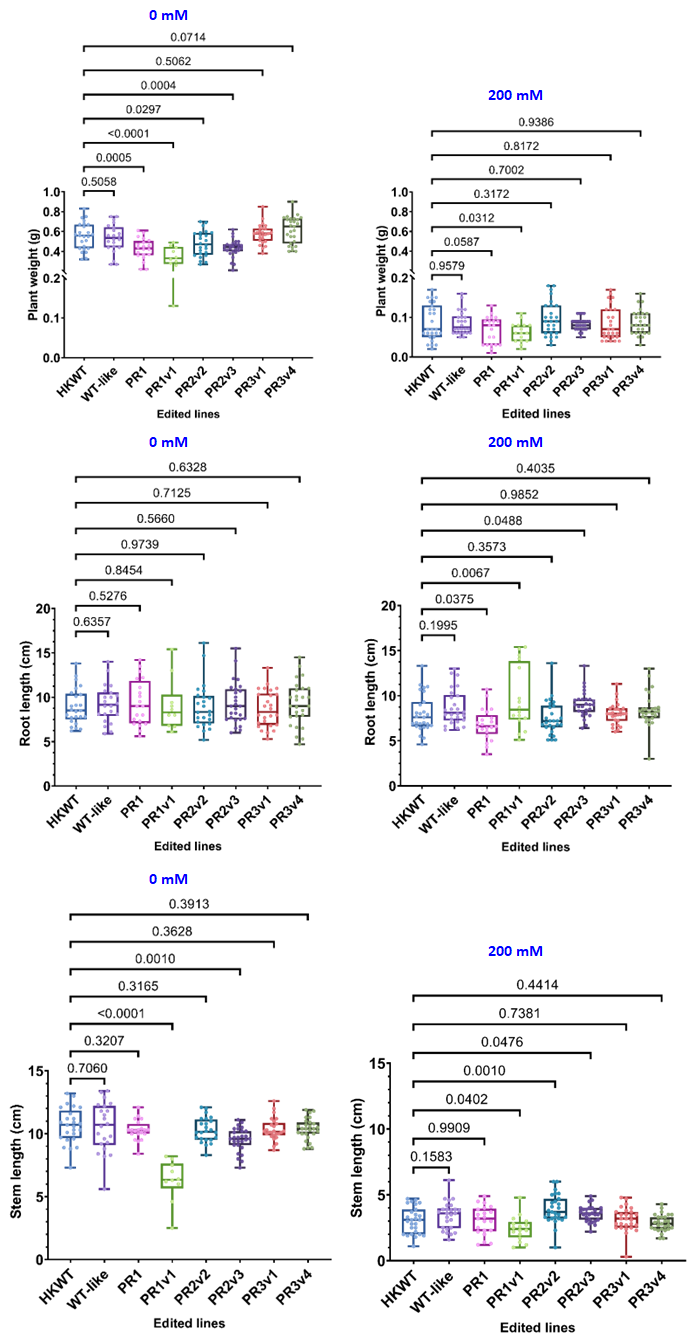


**Figure S11. The growth parameters of the edited lines, WT, and WT-like at 0 and 200 mM Mannitol.** The data were analyzed and plotted using Graphpad Prism 9.0. Data were collected from 10 to 24 biological replicates. The multiple comparisons of the relative expression levels were performed using One-way ANOVA uncorrected Fisher’s LSD test (P<0.05). The p-value is shown on the top of the compared boxes. HKWT: wild-type HK; WT-like: segregated WT siblings of the edited plants; PR1: precise edited event PR1; PR1v1: PR1 variant; PR2v2: variant 2 of precise edited event PR2; PR2v3: variant 3 of precise edited event PR2; PR3v1: variant 1 of precise edited event PR3; PR3v4: variant 4 of precise edited event PR3.


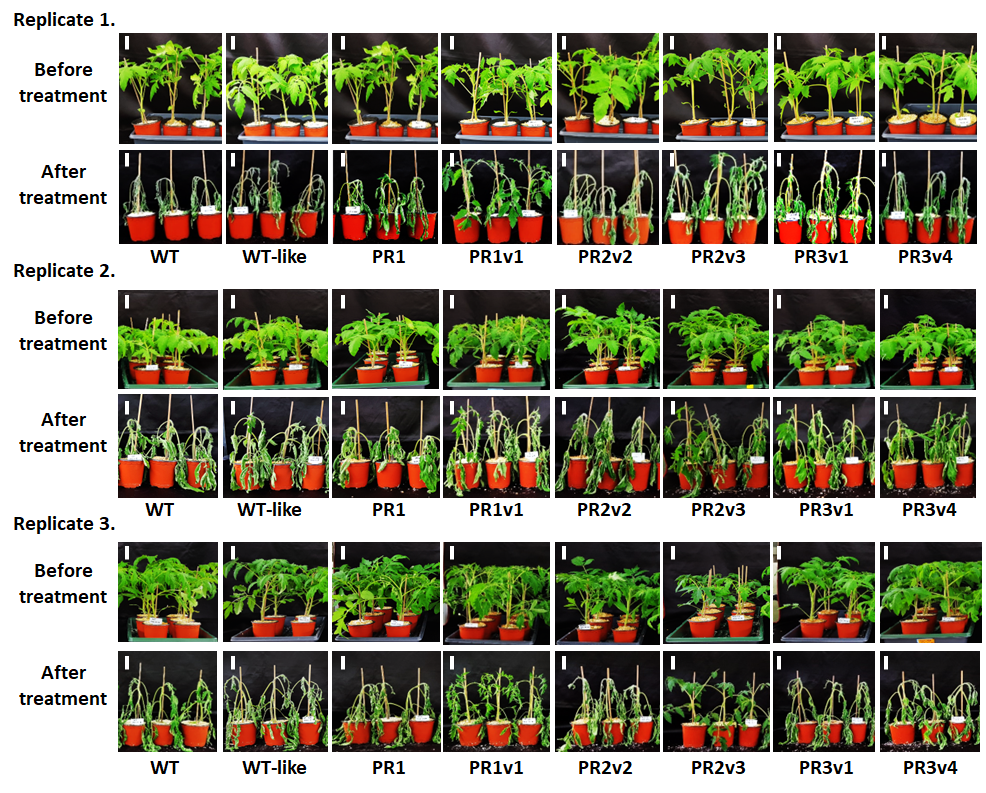


**Figure S12. Performance of the edited lines compared to the WT and WT-like plants (HK) after five days under drought stress.** Six-week-old seedlings were stopped watering and taken out all remaining water in the tray for five days and then watered to observe the recovery stage at 25^o^C (16h-light/8h-dark) in the culture room. Photographs were taken before treatment and five days after treatment. The name of plants/lines are denoted at the bottom of each panel. WT: wild-type HK; WT-like: segregated WT siblings of the edited plants; PR1, PR1v1; PR2v2; PR2v3; PR3v1 and PR3v4: edited lines. Scale bars: 2 cm.


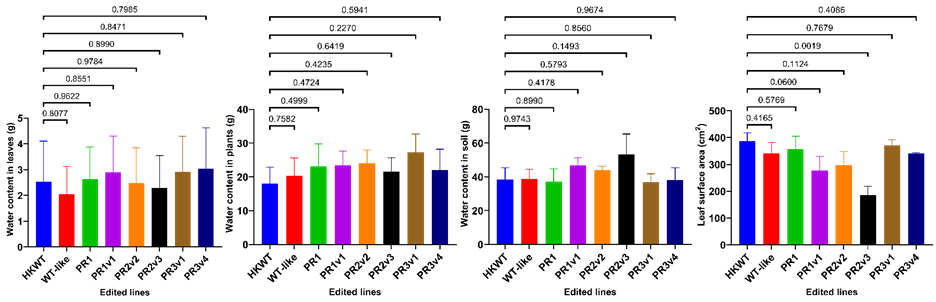


**Figure S13. Water content in leaves, plants, and soil and leaf surface area of *SlHyPRP1* edited lines (HK) under drought stress.** The data were analyzed and plotted using Graphpad Prism 9.0. Data were collected from three biological replicates. The multiple comparisons of the relative expression levels were performed using One-way ANOVA uncorrected Fisher’s LSD test (P<0.05). The p-value is shown on the top of the compared boxes. HKWT: wild-type HK; WT-like: segregated WT siblings of the edited plants; PR1: precise edited event PR1; PR1v1: PR1 variant; PR2v2: variant 2 of precise edited event PR2; PR2v3: variant 3 of precise edited event PR2; PR3v1: variant 1 of precise edited event PR3; PR3v4: variant 4 of precise edited event PR3.


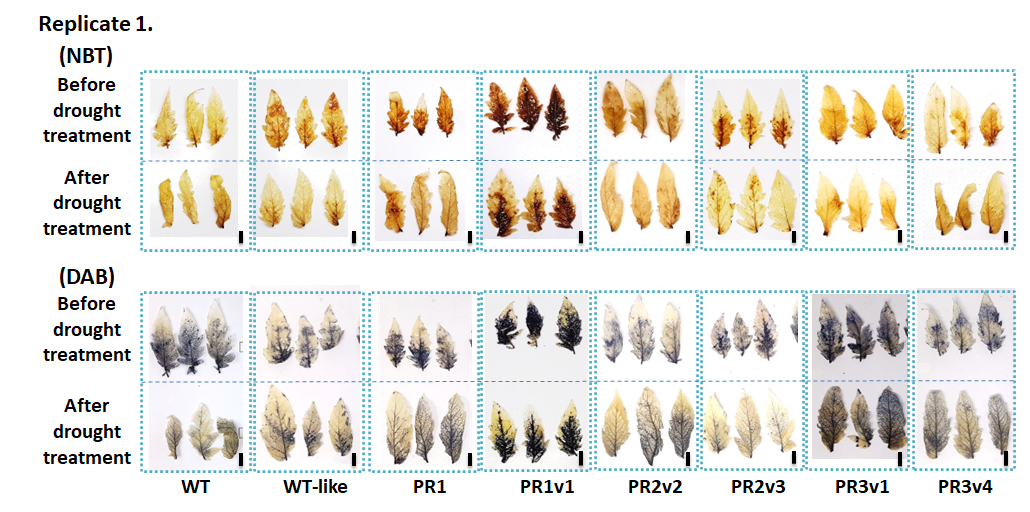


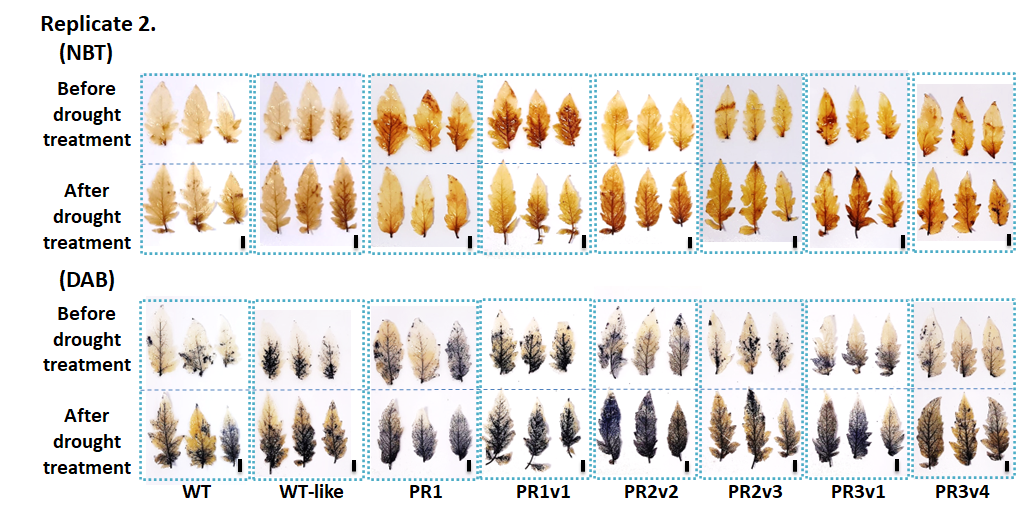


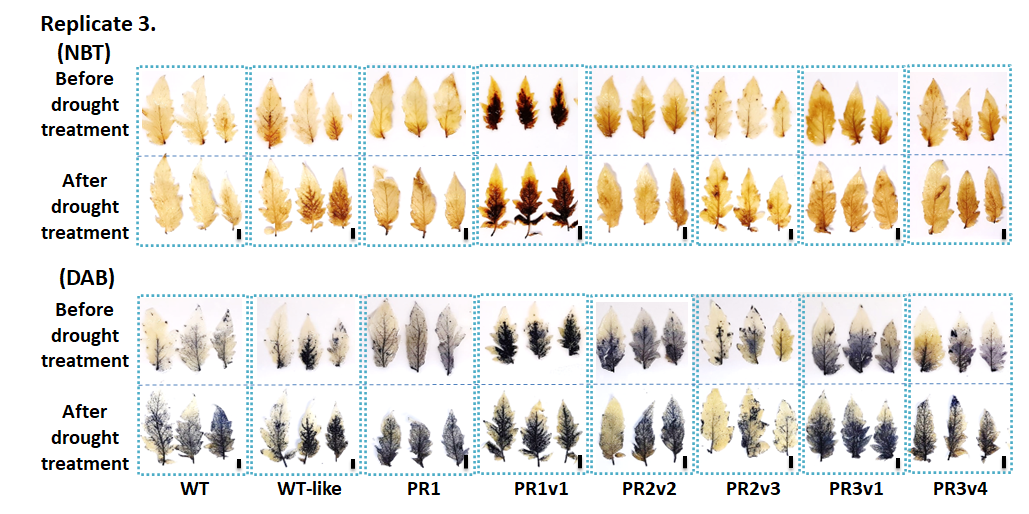


**Figure S14.** **Effects of the HK-based *SlHyPRP1* edited lines on ROS production (NBT and DAB staining) under drought stress.** Leaves of the drought-treated plants were collected before and after five days of the drought treatment and stained by the ROS-specific dyes. The top panel of each staining type represents leaves grown at 25°C before drought treatment, and the bottom panel represents leaves of the same plants that stopped watering for five days. WT: wild-type HK; WT-like: segregated WT siblings of the edited plants; PR1, PR1v1; PR2v2; PR2v3; PR3v1 and PR3v4: edited lines. Scale bars: 1 cm.

| **Table S1. Detailed data of the obtained alleles for heat and drought stress treatment** | | | | | | | | | | |
| --- | --- | --- | --- | --- | --- | --- | --- | --- | --- | --- |
| **No.** | **Event no.** | **Variety** | **Indel alleles** | **Generation** | **Construct (gRNA)** | **T-DNA insertion** | **Homozygous (hm)/ Heterozygous (ht)** | **Allele types** | **Salinity stress performance in the previous generation** | |
|  |  |  |  |  |  |  |  |  | **Germination stage** | **Growth stage** |
| 1 | 1011101-1508.06 | HK | PR1 | GE3 | pDR2 (gRNA1+gRNA2) | - | hm | SP-8CM | * | *** |
| 2 | 1021803-09 | 15T01 | PR1 | GE1 | pDR2 (gRNA1+gRNA2) | + | hm | SP-8CM | N/A | N/A |
| 3 | H127-2807.22 | HK | PR1v1 | GE3 | pDR1 (4 gRNAs) | - | hm | SP-8CM | *** | * |
|  |  |  |  |  |  |  |  | (Variant) |  |  |
| 4 | 1111003 | HK | PR2v1 | GE0 | pDR3 (gRNA3+gRNA4) | + | ht | SP-PRD | N/A | N/A |
|  |  |  |  |  |  |  |  | (Variant) |  |  |
| 5 | 1111021-0217 | HK | PR2v2 | GE2 | pDR3 (gRNA3+gRNA4) | - | hm | SP-PRD | * | * |
|  |  |  |  |  |  |  |  | (Variant) |  |  |
| 6 | H127-1507.11 | HK | PR2v3 | GE3 | pDR1 (4 gRNAs) | - | hm | SP-PRD | * | * |
|  |  |  |  |  |  |  |  | (Variant) |  |  |
| 7 | H20911 | 15T01 | PR3 | GE0 | pDR1 (4 gRNAs) | + | ht | SP | N/A | N/A |
| 8 | 1011116-1310 | HK | PR3v1 | GE2 | pDR2 (gRNA1+gRNA2) | - | hm | SP | ** | * |
|  |  |  |  |  |  |  |  | (Variant) |  |  |
| 9 | H21102 | 15T01 | PR3v2 | GE0 | pDR1 (4 gRNAs) | + | ht | SP | N/A | N/A |
| 10 | H20902-31 | 15T01 | PR3v3 | GE1 | pDR1 (4 gRNAs) | + | hm | (Variant) | N/A | N/A |
| 11 | 1011109-1114 | HK | PR3v4 | GE2 | pDR2 (gRNA1+gRNA2) | - | hm | SP | ** | * |
| 12 | 1011109-08 | HK | PR3v5 | GE1 | pDR2 (gRNA1+gRNA2) | - | hm | (Variant) | N/A | N/A |
| 13 | 1021803-0705 | 15T01 | PR3v6 | GE2 | pDR2 (gRNA1+gRNA2) | - | hm | SP | N/A | N/A |
| 14 | H20902-05 | 15T01 | PR3v7 | GE1 | pDR1 (4 gRNAs) | + | hm | (Variant) | N/A | N/A |
| *N/A: not yet assessed; *, **, and ***: low, medium, and high salinity tolerant levels, respectively.* | | | | | | | | | | |

**Table S2. Edited allele types showed multi-stress tolerance**

| **Allele Types** | **Precise/ variant editing** | **Allele no.** | **Variety** | **Multi-stress tolerance** | | | | | | |  |
| --- | --- | --- | --- | --- | --- | --- | --- | --- | --- | --- | --- |
|  |  |  |  | **Salinity** | | **Heat** | **Drought** | | **Pseudomonas** | **Fusarium** |  |
|  |  |  |  |  |  |  |  |  |  |  |  |
|  |  |  |  | **Germination stage** | **Growth stage** | **Growth stage** | **Germination stage** | **Growth stage** | **Growth stage** | **Growth stage** |  |
| **8CM** | Precise | PR1 | HK | **-** | **√** | **-** | **-** | **-** | **√** | **-** |  |
|  | Variant | PR1v1 | HK | **√** | **-** | **-** | **√** | **√** | **-** | **-** |  |
| **PRD** | Variants | PR2v2 | HK | **-** | **-** | **√** | **√** | **-** | **√** | **-** |  |
|  |  | PR2v3 | HK | **-** | **-** | **-** | **√** | **-** | **√** | **-** |  |
| **Full KO** | Variants | PR3v1 | HK | **√** | **-** | **√** | **-** | **-** | **-** | **-** |  |
|  |  | PR3v4 | HK | **√** | **-** | **√** | **-** | **-** | **-** | **-** |  |
|  |  | PR3v3 | 15T01 | **√** | **-** | **√** | **-** | **-** | **-** | **-** |  |
|  |  | PR3v7 | 15T01 | **√** | **-** | **√** | **-** | **-** | **-** | **-** |  |

**Table S3. Primer sequences used in this study**

| **No.** | **Primer name** | **Sequence (5’-3’)** | **Gene/allele name (locus name)** | **Binding sites** | **Product (bp)** |
| --- | --- | --- | --- | --- | --- |
| 1 | SlPDS-qF1 | GAGTCCAAGGTAGTTCAGCTTAT | *SlPDS* (Solyc03g123760) | Exon 1-exon 1 | 83 |
| 2 | SlPDS-qR1 | CCTTTGCAAGCAACCATCTC |  |  |  |
| 3 | SlHSP70-qF1 | CAAACTGAAAGAGCTCAAGG | Solyc11g020040.1 | | 100 |
| 4 | SlHSP70-qR1 | CTGTCCCAGCTGCATTACTT |  |  |  |
| 5 | SlHSFA1a-qF1 | AAATGATGTCGTTCCTGGC | Solyc08g005170 | | 115 |
| 6 | SlHSFA1a-qR1 | ATCCTTCGTTTCTTGCTGC |  |  |  |
| 7 | SlCAT1-qF1 | GGTGGATTATTTGCCCTCG | Solyc12g094620.1.1 | | 137 |
| 8 | SlCAT1-qR1 | ACCTCTCCCCTGCCTGTTT |  |  |  |
| 9 | SlCu/ZnSOD-qF1 | CCGACAAGCAGATTCCTCTC | Solyc01g067740.2.1 | | 97 |
| 10 | SlCu/ZnSOD-qR1 | TCATGTCCTCCCTTTCCAAG |  |  |  |
| 11 | SlHyPRP1-Fwd1 | GCATTCAACAATCATCAAGTACTCA | Solyc12g009650.1.1 | | 1000 |
| 12 | SlHyPRP1-Rev1 | CAGTAGTACGACGGGTGTTTAAT |  |  |  |
| 13 | SlHyPRP1-Fwd1 | GCATTCAACAATCATCAAGTACTCA |  |  | 1606 |
| 14 | SlHyPRP1-Rev2 | AACAATTCCACAAAGCCAAA |  |  |  |
| 15 | SlHyPRP1CDS1-F1 | CAGTCGAAGACAAAATGATGGAGTTC  TCTAAGATAACTTCACTTCTT |  |  | 1541 |
| 16 | SlHyPRP1-Rev2 | AACAATTCCACAAAGCCAAA |  |  |  |
| 17 | NptII-F5 | TGGAGAGGCTATTCGGCTATG |  | | 2175 |
| 18 | 35S-R3 | CGTCAGTGGAGATGTCACATCA |  |  |  |
| 19 | GAPDH-F1 | CCATAACCTAATTTCTCTCTC | *SlGAPDH* (Solyc05g014470.2) | | 1073 |
| 20 | GAPDH-R1 | GTCATGAGACCCTCAACAAT |  |  |  |
| 21 | SlACT-qF | GAAATAGCATAAGATGGCAGACG | Solyc04g011500.3.1 | | 157 |
| 22 | SlACT-qR | ATACCCACCATCACACCAGTAT |  |  |  |
| 23 | SlGAPDH-qF | CTGCTCTCTCAGTAGCCAACAC | Solyc04g009030.2 | | 157 |
| 24 | SlGAPDH-qR | CTTCCTCCAATAGCAGAGGTTT |  |  |  |
| 25 | SlMYC2-qF | agcaggagcatcggaagaa | Solyc08g076930 | | 191 |
| 26 | SlMYC2-qR | ccaaatcgggctggaacta |  |  |  |
| 27 | SlPR1-qF | GCCATATTTCACTCTTGTGAG | Solyc09g007010.1 | | 257 |
| 28 | SlPR1-qR | TAGTCTGGCCTCTCGGACA |  |  |  |
| 29 | SlPR2-qF | TCCAGGTAGAGACAGTGGTAAA | Solyc01g008620.2 | | 142 |
| 30 | SlPR2-qR | GTAGGTGTTGGTTAAAAGCCC |  |  |  |
| 31 | TomLoxD-qF | CGTCCTAAACTTGGAGGAGAAG | Solyc03g122340.2 | | 145 |
| 32 | TomLoxD-qR | CCTCAAATTGTTCATCTCTCGG |  |  |  |
